# Synthesis, structure, spectroscopy and reactivity of new heterotrinuclear water oxidation catalysts†
†Electronic supplementary information (ESI) available: Complete experimental procedures, compound characterization data and experimental data. CCDC 1440794 and 1440795. For ESI and crystallographic data in CIF or other electronic format see DOI: 10.1039/c5sc04672f


**DOI:** 10.1039/c5sc04672f

**Published:** 2016-02-02

**Authors:** Lorenzo Mognon, Sukanta Mandal, Carmen E. Castillo, Jérôme Fortage, Florian Molton, Guillem Aromí, Jordi Benet-Buchhlolz, Marie-Noëlle Collomb, Antoni Llobet

**Affiliations:** a Institute of Chemical Research of Catalonia (ICIQ) , Barcelona Institute of Science and Technology , Avinguda Països Catalans 16 , 43007 Tarragona , Spain . Email: allobet@iciq.cat; b Department of Chemistry , Indian Institute of Technology Kharagpur , Kharagpur-721302 , West Bengal , India; c Univ. Grenoble Alpes , DCM , F-38000 Grenoble , France; d CNRS , DCM , F-38000 Grenoble , France; e Departament de Química Inorgànica , Universitat de Barcelona , Diagonal 645 , 08028 Barcelona , Spain; f Departament de Química , Universitat Autònoma de Barcelona , Cerdanyola del Vallès , 08193 Barcelona , Spain

## Abstract

Four heterotrinuclear complexes containing the ligands 3,5-bis(2-pyridyl)pyrazolate (bpp^–^) and 2,2′:6′,2′′-terpyridine (trpy) of the general formula {[Ru^II^(trpy)]_2_(μ-[M(X)_2_(bpp)_2_])}(PF_6_)_2_, where M = Co^II^, Mn^II^ and X = Cl^–^, AcO^–^ (M = Co^II^, X = Cl^–^: **Ru_2_Co–Cl_2_**; M = Mn^II^, X = Cl^–^: **Ru_2_Mn–Cl_2_**; M = Co^II^, X = AcO^–^: **Ru_2_Co–OAc_2_**; M = Mn^II^, X = AcO^–^: **Ru_2_Mn–OAc_2_**), have been prepared for the first time. The complexes have been characterized using different spectroscopic techniques such as UV-vis, IR, and mass spectrometry. X-Ray diffraction analyses have been used to characterize the **Ru_2_Mn–Cl_2_** and **Ru_2_Mn–OAc_2_** complexes. The cyclic voltammograms (CV) for all four complexes in organic solvent (CH_3_CN or CH_2_Cl_2_) display three successive reversible oxidative waves corresponding to one-electron oxidations of each of the three metal centers. The oxidized forms of the complexes **Ru_2_Co–OAc_2_** and **Ru_2_Mn–OAc_2_** are further characterized by EPR and UV-vis spectroscopy. The magnetic susceptibility measurements of all complexes in the temperature range of 2–300 K reveal paramagnetic properties due to the presence of high spin Co(ii) and Mn(ii) centers. The complexes **Ru_2_Co–OAc_2_** and **Ru_2_Mn–OAc_2_** act as precatalysts for the water oxidation reaction, since the acetato groups are easily replaced by water at pH = 7 generating the active catalysts, {[Ru(H_2_O)(trpy)]_2_(μ-[M(H_2_O)_2_(bpp)_2_])}^4+^ (M = Co^II^: **Ru_2_Co–(H_2_O)_4_**; M = Mn^II^: **Ru_2_Mn–(H_2_O)_4_**). The photochemical water oxidation reaction is studied using [Ru(bpy)_3_]^2+^ as the photosensitizer and Na_2_S_2_O_8_ as a sacrificial electron acceptor at pH = 7. The Co containing complex generates a TON of 50 in about 10 minutes (TOF_i_ = 0.21 s^–1^), whereas the Mn containing complex only generates a TON of 8. The water oxidation reaction of **Ru_2_Co–(H_2_O)_4_** is further investigated using oxone as a sacrificial chemical oxidant at pH = 7. Labelled water oxidation experiments suggest that a nucleophilic attack mechanism is occurring at the Co site of the trinuclear complex with cooperative involvement of the two Ru sites, *via* electronic coupling through the bpp^–^ bridging ligand and *via* neighboring hydrogen bonding.

## Abstract 2

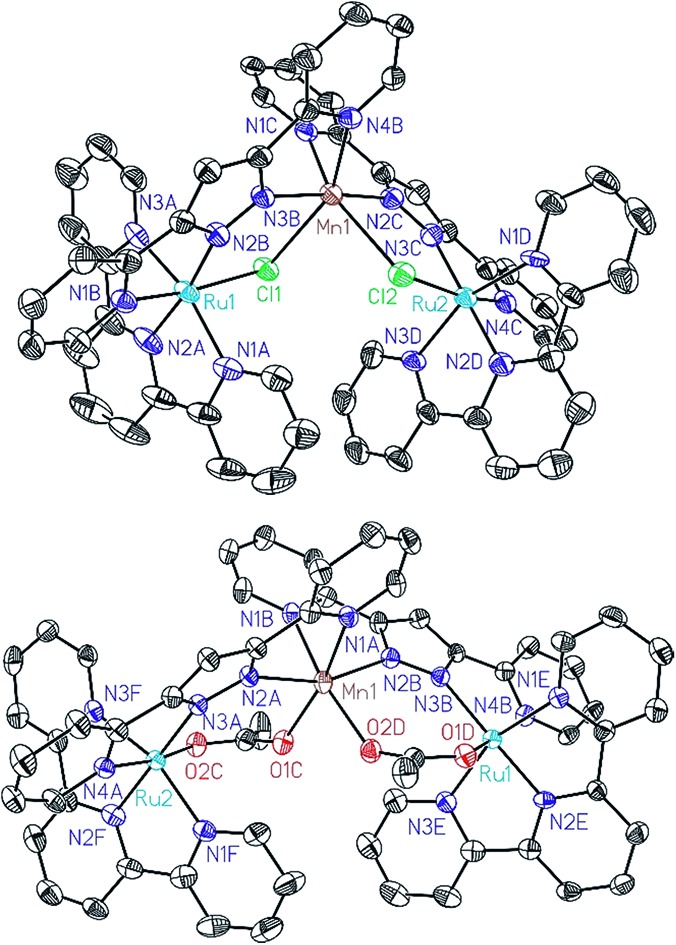
Four heterotrinuclear complexes containing the ligands 3,5-bis(2-pyridyl)pyrazolate (bpp^–^) and 2,2′:6′,2′′-terpyridine (trpy) of the general formula {[Ru^II^(trpy)]_2_(μ-[M(X)_2_(bpp)_2_])}(PF_6_)_2_ have been prepared for the first time.

## Introduction

Water oxidation catalysis is a field that has expanded enormously over the last few years.^1^–^10^ This development has been fuelled by interest in the topic for the achievement of new energy conversion schemes based on water and sunlight to obtain so-called solar fuel (such as H_2_), mimicking natural photosynthesis. Water oxidation is the main reaction that occurs at the OEC-PSII,^11^ yielding molecular oxygen, four protons and four electrons, which can later react further. The development of new water oxidation catalysts (WOCs) has been based mainly on mononuclear^12^ and dinuclear^13^,^14^ Ru complexes containing polypyridilic ligands. However, recently, a number of Ir and first row transition metal complexes have also been reported to be able to oxidize water.^3^,^15^,^16^

While mononuclear complexes are in general easier to prepare, targeted polynuclear complexes can present significant synthetic challenges. Nevertheless, polynuclear complexes containing bridging ligands that can electronically couple the metal centers can attain important benefits from the perspective of a WOC. For example, multiple electronically coupled redox active metal centers can cooperate during the four electron transfers needed for the water oxidation reaction. On the other hand, non-redox active centers can be of interest to exert electronic perturbation over the redox active center.^17^,^18^ They can also provide aqua/hydroxo ligands which, when strategically situated, can help to lower activation energies by hydrogen bonding with key active species bonded to the active metal, or even participate in O–O bond formation, as has been proposed for the Ca–OH_2_ moiety of the OEC-PSII on a number of occasions.^19^,^20^

With these considerations in mind, we undertook the preparation of heterotrinuclear complexes where all of the metal centers are redox active and possess aqua ligands, to favor the achievement of high oxidation states *via* Proton Coupled Electron Transfer (PCET). Herein, we report the synthesis, structural, spectroscopic and electrochemical characterization of a new family of heterotrinuclear complexes containing Ru and Co or Mn as metal centers, together with their capacity to oxidize water to dioxygen.

## Results and discussion

### Synthesis and solid state structure

**Scheme 1:**
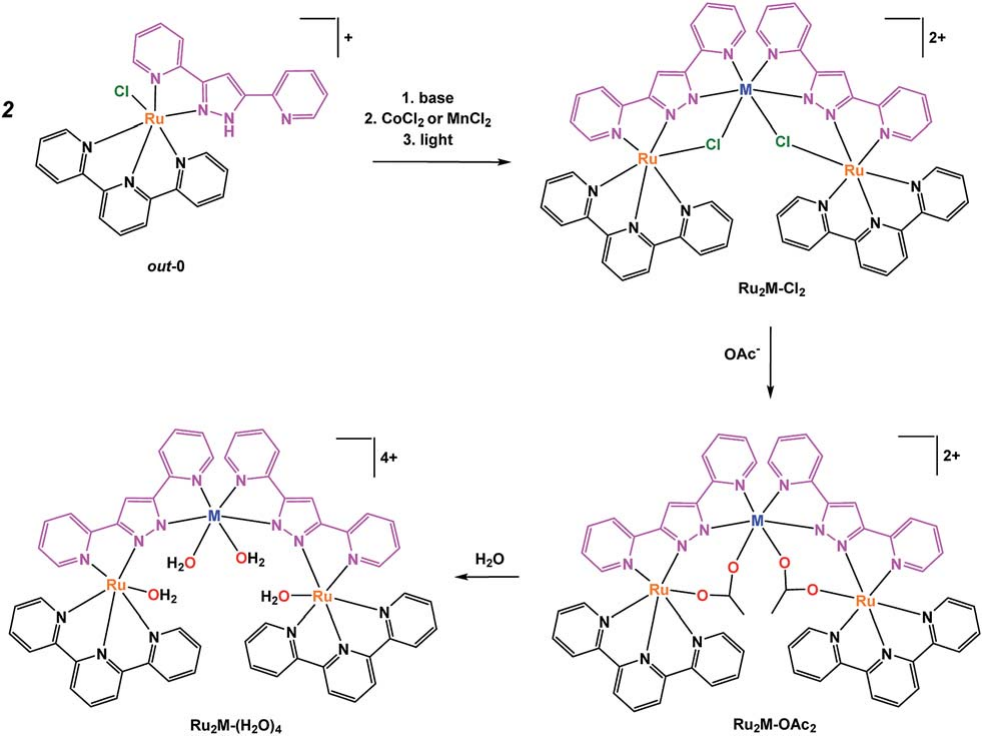
Synthetic strategy followed for the preparation of trinuclear complexes and their labelling.

The well-known Hbpp ligand is an asymmetric molecule which, once deprotonated, presents two equivalent coordination environments. The protonated ligand thus allows the preparation of mononuclear complexes that can be used as starting materials for the preparation of homo-21 or heterodinuclear, or heterotrinuclear^22^ complexes. In the present report we use *out*-[Ru(Cl)(Hbpp)(trpy)]^+^, ***out*-0**, as the starting material for the preparation of heterotrinuclear complexes containing redox active metals, as displayed in Scheme 1.

The pyrazolato proton of ***out*-0** is removed using NaOMe as a base, and then MnCl_2_ or CoCl_2_ salts are used to react with the vacant coordination sites of the bpp^–^ ligand, as shown in eqn (1) for the case of cobalt.
1

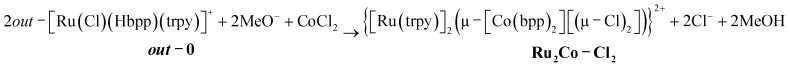




Complex **Ru_2_Co–Cl_2_** is obtained in 58% yield, whereas a 70% yield is obtained for **Ru_2_Mn–Cl_2_** following a similar synthetic procedure.^22^ Treatment of these trinuclear complexes with sodium acetate at 75 °C in an acetone : water (5 : 1) solution replaces the chlorido bridging ligands by the more labile acetato ligands, as indicated in the following equation.
2

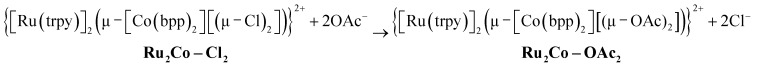




Finally, substitution of the acetato bridges by monodentate aqua ligands leads to the formation of the corresponding tetra-aqua complex in neutral pH (eqn (3)).
3

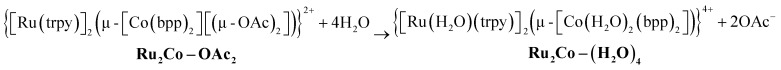




In contrast, under acidic conditions, the trinuclear complexes decompose to the corresponding mononuclear *in*-[Ru(Hbpp)(trpy)(H_2_O)]^2+^, ***in*-1**, and free Co(ii) (Fig. S27†).

The synthetic strategies used for the preparation of **Ru_2_Mn–OAc_2_**and **Ru_2_Mn–(H_2_O)_4_** are analogous to those for the cobalt counterparts.

**Fig. 1:**
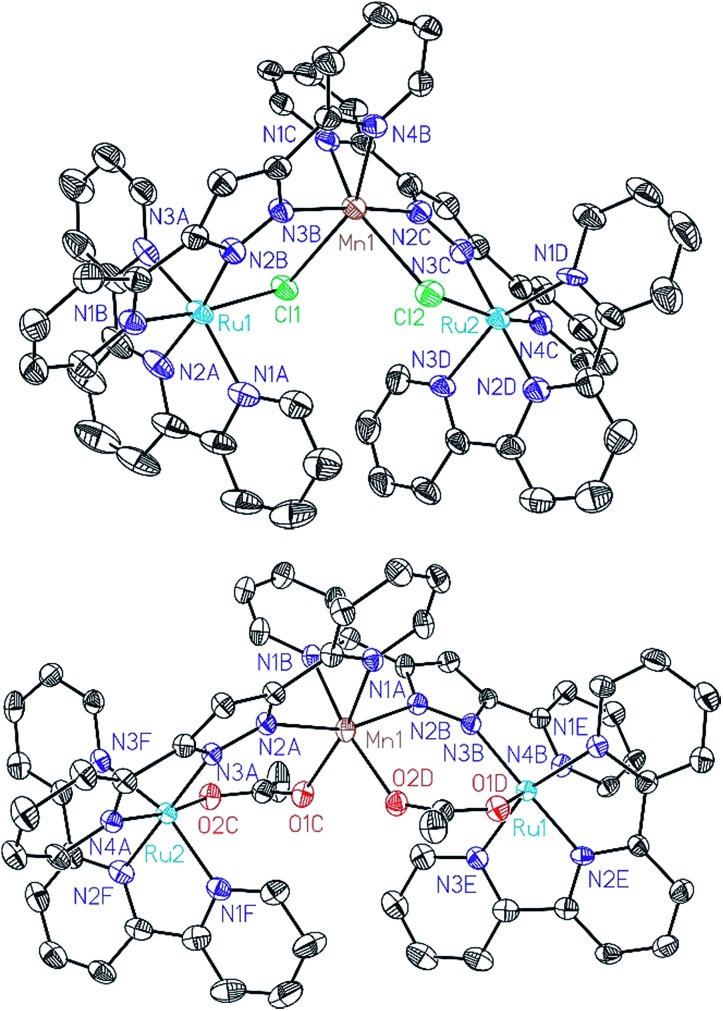
ORTEP plots (50% probability) for the cationic structures of complexes **Ru_2_Mn–Cl_2_** (top) and **Ru_2_Mn–(OAc)_2_** (bottom). Hydrogen atoms have been omitted.

All of these new complexes have been characterized by analytic, spectroscopic or electrochemical techniques. Further, X-ray diffraction analysis has been carried out for the Mn complexes, **Ru_2_Mn–Cl_2_** and **Ru_2_Mn–OAc_2_**, and ORTEP views of their cationic moiety are presented in Fig. 1.

All the metal centers present pseudo-octahedral symmetry around the first coordination sphere. In the **Ru_2_Mn–Cl_2_** case, the ruthenium atoms are coordinated by five N-atoms, three from a meridional trpy ligand and two from bpp^–^, while the sixth coordination position is occupied by a chlorido ligand. The manganese center is coordinated by the same two bridging chlorido moieties, in a *cis* fashion, and by the two chelating bpp^–^ ligands. The “Mn(bpp)_2_” moiety can thus be considered as a bridge between the two Ru centers (see Fig. 1 and Scheme 1). A very similar structure is obtained for **Ru_2_Mn–OAc_2_** where the bridging chlorido ligands have been substituted by bridging acetato ligands. For both structures, the bonding distances and angles presented by the Ru(ii) and Mn(ii) centers are unremarkable.^23^,^24^

### Redox properties and UV-vis spectroscopy in organic solvents for **Ru_2_M–OAc_2_**

**Fig. 2:**
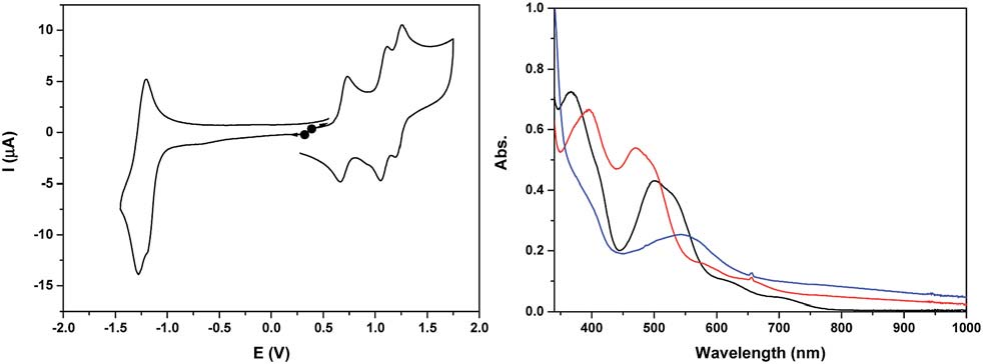
(Left) Cyclic voltammogram of a 0.25 mM solution of **Ru_2_Co–OAc_2_** in 0.1 M [(*n*Bu_4_N)ClO_4_] in CH_3_CN, at a scan rate of 100 mV s^–1^. (Right) UV-visible absorption spectra of a 0.25 mM solution of **Ru_2_Co–OAc_2_** in 0.1 M [(*n*Bu_4_N)ClO_4_] in CH_3_CN, in oxidation states (black) Ru_2_(ii)Co(ii), (red) Ru_2_(ii)Co(iii) and (blue) Ru_2_(iii)Co(iii).

The cyclic voltammogram of **Ru_2_Co–OAc_2_** in CH_3_CN (Fig. 2) displays three successive reversible oxidation waves at *E*_1/2_ = 0.70 (Δ*E* = 75 mV), 1.08 (Δ*E* = 60 mV), and 1.22 V (Δ*E* = 60 mV), and one reversible reduction wave at *E*_1/2_ = –1.23 V (Δ*E* = 80 mV). All the redox potentials in this work are reported *vs.* NHE. Each of the three oxidation processes corresponds to the exchange of one electron per molecule of complex, as evidenced by rotating disk electrode experiments (Fig. S19†). The first process is assigned to the oxidation of the cobalt Co(iii)/Co(ii) and the last two to the oxidation of the two Ru sites, Ru_2_(ii,iii)/Ru_2_(ii,ii) and Ru_2_(iii,iii)/Ru_2_(ii,iii). The presence of two distinct one-electron redox processes, close in potential (Δ*E*_1/2_ = 140 mV), instead of a two-electron single wave, is in agreement with there being two identical electroactive centers in the molecule that can electronically communicate.^24^ This is the case of the two Ru sites interacting through the conjugation of the bridging bpp^–^ ligands and the acetato bridge *via* the central Co^3+^ core. The two electron reduction wave at –1.23 V is assigned to the reduction of the terpyridine units of the Ru(ii) centers. The shoulder observed at –1.18 V indicates that the two terpyridine ligands are not fully equivalent, which is in accordance with there being weak electronic coupling between the two Ru subunits.

The two oxidized forms of the complex, Ru_2_(ii)Co(iii) and Ru_2_(iii)Co(iii) are stable in CH_3_CN, as tested by successive electrolysis at *E* = 0.85 V and *E* = 1.40 V, which consume, respectively, one and two electrons (Fig. S18†). Rotating disk electrode experiments confirm the quantitative formation of these species (Fig. S19†). The electrogenerated solutions have also been analyzed by UV-visible (Fig. 2) and EPR spectroscopy (Fig. 4, see below).

The three stable oxidation states, Ru_2_(ii)Co(ii), Ru_2_(ii)Co(iii) and Ru_2_(iii)Co(iii), have distinct UV-visible signatures. The initial orange Ru_2_(ii)Co(ii) solution exhibits two intense visible bands at 366 and 500 nm (with a shoulder at 530 nm) and two less intense shoulders at 620 and 710 nm. The oxidation of the central Co(ii) unit into Co(iii) leads to a shift of the intense visible bands to 396 and 473 nm (shoulder at 500 nm) and of the two shoulders to 580 and 655 nm. A more pronounced color change of the solution is observed when the two Ru(ii) species are oxidized into Ru(iii), as indicated by the replacement of all the previous visible bands by new ones at 390 (shoulder) and 545 nm. An increase in absorption is also observed between 600 and 1000 nm. As the more evident changes occur after the oxidation from Ru_2_(ii)Co(iii) to Ru_2_(iii)Co(iii), the intense visible absorption bands originate from the ruthenium units.

Back electrolysis of the final solution conducted at 0.35 V (three electrons exchanged) restores the initial complex, Ru_2_(ii)Co(ii), quantitatively (Fig. S20†). This demonstrates the perfect stability of the different oxidation states of the trinuclear compound, and the reversibility of the processes.

**Fig. 3:**
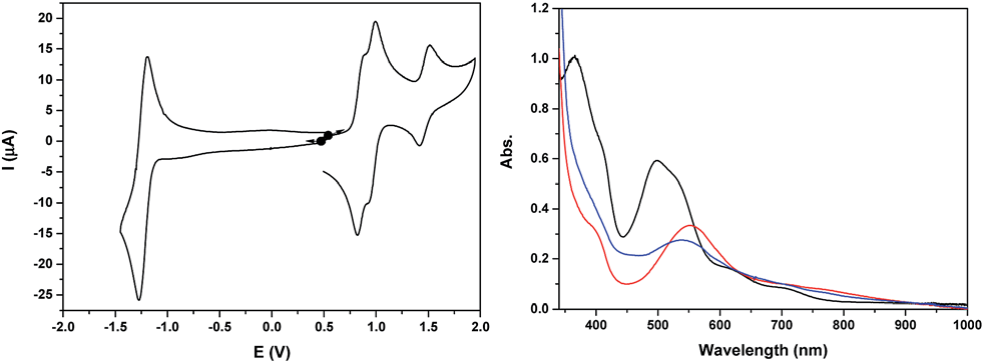
(Left) Cyclic voltammogram of a 0.41 mM solution of **Ru_2_Mn–OAc_2_** in 0.1 M [(*n*Bu_4_N)ClO_4_] in CH_3_CN, at a scan rate of 50 mV s^–1^. (Right) UV-visible absorption spectra of a 0.41 mM solution of **Ru_2_Mn–OAc_2_** in 0.1 M [(*n*Bu_4_N)ClO_4_] in CH_3_CN, in oxidation states, (black) Ru_2_(ii)Mn(ii), (red) Ru_2_(iii)Mn(ii) and (blue) Ru_2_(iii)Mn(iii).

The cyclic voltammogram of **Ru_2_Mn–OAc_2_** in CH_3_CN (Fig. 3 and S21†) also displays three successive one-electron reversible oxidation waves at *E*_1/2_ = 0.85 V (Δ*E* = 60 mV), 0.96 V (Δ*E* = 70 mV) and 1.47 V (Δ*E* = 100 mV) at a scan rate of 50 mV s^–1^ and a reversible two-electron terpyridine-centered reduction wave at *E*_1/2_ = –1.23 V (Δ*E* = 80 mV). The two first oxidation waves, very close in potential (Δ*E*_1/2_ = 110 mV), are thus assigned to the oxidation of the Ru sites, Ru_2_(ii,iii)/Ru_2_(ii,ii) and Ru_2_(iii,iii)/Ru_2_(ii,iii) and the last one to the Mn central unit, Mn(iii)/Mn(ii).

**Fig. 4:**
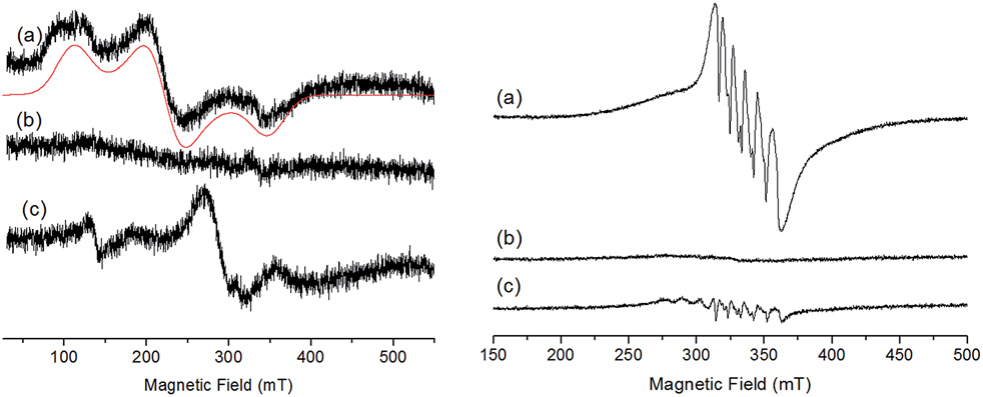
(Left) X-band EPR spectra recorded at 13 K of a 0.25 mM solution of **Ru_2_Co–OAc_2_** in 0.1 M [(*n*Bu_4_N)ClO_4_] in CH_3_CN, in oxidation states (a) Ru_2_(ii)Co(ii), (b) Ru_2_(ii)Co(iii) and (c) Ru_2_(iii)Co(iii) (black), and corresponding simulated spectra (red). (Right) X-band EPR at 100 K of a 0.41 mM solution of **Ru_2_Mn–OAc_2_** in 0.1 M [(*n*Bu_4_N)ClO_4_] in CH_3_CN, in oxidation states (a) Ru_2_(ii)Mn(ii), (b) Ru_2_(iii)Mn(ii) and (c) Ru_2_(iii)Mn(iii).

The stability of the two oxidized states, Ru_2_(iii)Mn(ii) and Ru_2_(iii)Mn(iii), has been evaluated by two successive electrolyses at *E* = 1.11 V and 1.69 V. The first electrolysis at 1.11 V consumed two electrons per molecule of initial complex and lead to the quantitative formation of the Ru_2_(iii)Mn(ii) species. An additional one electron oxidation carried out at 1.69 V leads to the formation of the fully oxidized form, Ru_2_(iii)Mn(iii). At this stage, the presence of an additional reversible process at *E*_1/2_ = 1.23 V with very small intensity should be pointed out, which is probably related to minor decomposition of the complex by decoordination of the Mn ion (less than 5%, Fig. S21†). Indeed, the potential of this new process is similar to that of a RuN_6_ mononuclear complex such as *in*-[Ru(Hbpp)(trpy)(CH_3_CN)]^2+^. Both electrolysis processes have been monitored by UV-vis and X-band EPR spectroscopy (Fig. 3 and 4, respectively).

The UV-vis absorption spectrum of **Ru_2_Mn–OAc_2_**, with two intense visible bands at 365 and 497 nm (with a shoulder at 530 nm) and two less intense shoulders at 620 and 710 nm, is nearly identical to that of **Ru_2_Co–OAc_2_** (see Fig. S9† for a comparison of the spectra). These observations confirm that the visible absorption bands originate mainly from the Ru units. Once the two Ru(ii) units have been oxidized, formation of the Ru_2_(iii)Mn(ii) species leads to significant changes with the replacement of the initial visible bands by new ones at 400 (shoulder) and 553 nm and two shoulders at 710 and 764 nm. For the fully oxidized solution, the oxidation of the central Mn(ii) unit into Mn(iii) leads to minor changes, with a shift of the band at 553 nm to 538 nm and a small increase of the absorption around 450 nm (Fig. 3).

The initial reduced state of the complex **Ru_2_Mn–OAc_2_** is restored almost quantitatively by a back electrolysis of the final solution at 0.35 V (Fig. S22†).

### EPR properties

The X-band EPR spectra of the initial and electrochemically oxidized solutions of **Ru_2_Co–OAc_2_** have been recorded at low temperature (13 K) (Fig. 4). The initial solution of **Ru_2_Co–OAc_2_** shows an EPR signal characteristic of a Co(ii) ion (d^7^) in the high spin state (*S* = 3/2),^25^ arising from the central Co(ii) unit, as both Ru units (Ru(ii), d^6^) are diamagnetic and thus EPR silent. The analysis of high spin Co(ii) centers is difficult because the zero field splitting (ZFS) energy is usually greater than the Zeeman interaction, leading to spectra insensitive to the magnitude of the axial term (*D*) of the ZFS. Consequently, only the real *g*-values (*g*_real_) and the rhombicity (*E*/*D* ratio) of the system can be extracted from the simulation of the EPR spectra. Simulation of the experimental data provides the following spin-Hamiltonian parameters for the *M*_s_ = |±1/2 = |±1/2〉: : *g*_real_(*x*, *y*) = 2.42, *g*_real_(*z*) = 2.31, and *E*/*D* = 0.23.

After the one-electron oxidation into Ru_2_(ii)Co(iii), the solution becomes EPR silent, in accordance with the formation of the low-spin Co(iii) species (d^6^) (*S* = 0).^26^

The EPR spectrum of the fully oxidized solution of Ru_2_(iii)Co(iii) displays two features ascribable to an effective *S* = 1 spin state which results from a magnetic interaction between two *S* = 1/2 spin states located on the Ru(iii) metals. The characteristic rhombic signature of the low spin Ru(iii) *S* = 1/2 spin state (d^5^) between 210 and 360 mT is retained in the spectrum.^27^,^28^ A weak half-field EPR line is also observed at 139 mT, which is consistent with the *S* = 1 spin state induced by the magnetic interaction of two Ru(iii) ions. EPR spectra with similar features have been previously reported for magnetically coupled dinuclear Ru(iii)–Ru(iii) complexes.^28^

The EPR signal of the initial **Ru_2_Mn–OAc_2_** solution at 100 K exhibits a 6-line signal characteristic of a high-spin Mn(ii) ion (3d^5^, *S* = 5/2), related to the central Mn(ii) unit of the complex (Fig. 4).^27^ This signal fully disappears after the oxidation of the two diamagnetic Ru(ii) units into Ru(iii). Although an EPR signal is expected for a magnetically coupled system involving two Ru(iii) (*S* = 1/2) ions and one Mn(ii) (*S* = 5/2), no EPR signal was detected regardless of temperature (from 13 K to 200 K), presumably due to fast relaxation. The fully oxidized solution of Ru_2_(iii)Mn(iii) is also EPR silent, which is in agreement with there being magnetic coupling between the two Ru(iii) (*S* = 1/2) and the Mn(iii) (d^4^, *S* = 2) ions, which must produce a integer spin ground state. The low-intensity signals observed around *g* = 2 are attributed to some decomposition of the trinuclear Ru_2_(iii)Mn(iii) (OAc)_2_ complex (less than 5%, as shown by electrochemistry) into “free” Mn(ii) (six line feature centered at *g* = 2.0, 339 mT) and the mononuclear [Ru^III^(bpp)(trpy)(CH_3_CN)]^3+^ complex (*S* = 1/2) (the two features at a low magnetic field compared to the six line feature).

### Magnetic properties

Magnetic susceptibility measurements were performed under a constant magnetic field of 5000 Oe, in the temperature range of 2–300 K. Variable field reduced magnetization at 2 K was performed for all complexes. Isofield variable temperature reduced magnetization measurements were also performed under various fields. These experiments served as criteria of purity, since this chemistry often leads to the formation of small impurities of Ru(0) particles that become very visible when the magnetic properties are examined.

**Fig. 5:**
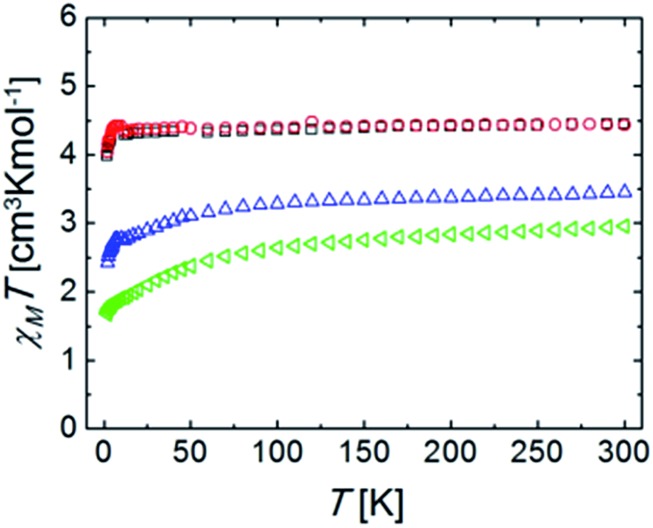
Plots of *χT vs. T* (*χ* is the molar paramagnetic susceptibility) for complexes (blue) **Ru_2_Co–Cl_2_**, (red) **Ru_2_Mn–Cl_2_**, (green) **Ru_2_Co–OAc_2_**, and (black) **Ru_2_Mn–OAc_2_**, under a constant magnetic field of 5000 Oe.

The constant field and variable temperature magnetic susceptibility results are represented in the form of *χT vs. T* curves (where *χ* is the molar paramagnetic susceptibility) (Fig. 5).

The *χT* products for the complexes **Ru_2_Co–Cl_2_** and **Ru_2_Co–OAc_2_** are, at 300 K, 3.44 and 2.96 cm^3^ K mol^–1^, respectively, which are significantly higher than the expected values for spin-only Co(ii) ions (calculated as 1.875 cm^3^ K mol^–1^ for *g* = 2.0 and *S* = 3/2). This product decreases with an increasing rate as the temperature declines. Both observations are accounted for by the fact that octahedral high spin Co(ii) exhibits an orbital angular momentum (*L* = 1) in addition to the spin state (*S* = 3/2). This causes the observation of an effective *g* value that is much higher than expected for a spin only system (here calculated as 2.51 and 2.71, respectively). The differences between both ions are caused by their slightly differing coordination geometries, as well as the varying ligand field strength of some of the donors.^29^ The depopulation of the *J* multiplets arising from the spin–orbit coupling explains the decline of *χT* upon cooling. The reduced magnetization curves measured at 2 K (Fig. S25†) saturate for both compounds at values lower than expected for an *S* = 3/2 system. This is because at such a low temperature, only the lowest *J* multiplet is populated. Isofield magnetization curves (at temperatures below 7 K and under several fields) produce the same saturation values and show quasi-superposition of the various curves (Fig. S26†). This shows that the anisotropy of these low energy states is very small. These results are consistent with the above characterization and with the interpretation of the EPR results.

At 300 K, the *χT* products for the complexes **Ru_2_Mn–Cl_2_** and **Ru_2_Mn–OAc_2_** are almost identical; 4.43 and 4.45 cm^3^ K mol^–1^, respectively, which correspond to the expected spin-only value for an isolated Mn(ii) center with *S* = 5/2 spin state and *g* = 2.01 and 2.02, respectively, consistent with the EPR and electrochemical results. This value stays nearly constant until around 5 K, where a decline down to 3.98 and 4.04 cm^3^ K mol^–1^ at 2 K, respectively, is observed. The low temperature decline can be due to a small value of ZFS, or weak antiferromagnetic intermolecular interactions. The variable field reduced magnetization curves, measured at 2 K (Fig. S23†), and the isofield magnetization curves (Fig. S24†) are the result of Mn(ii) saturating near 5 Bohr magneton, and this is consistent with the above results. The fact that the isofield lines superimpose with each other for the various fields employed confirms that the amount of ZFS is very small for both complexes.

### Redox properties in aqueous solutions

**Fig. 6:**
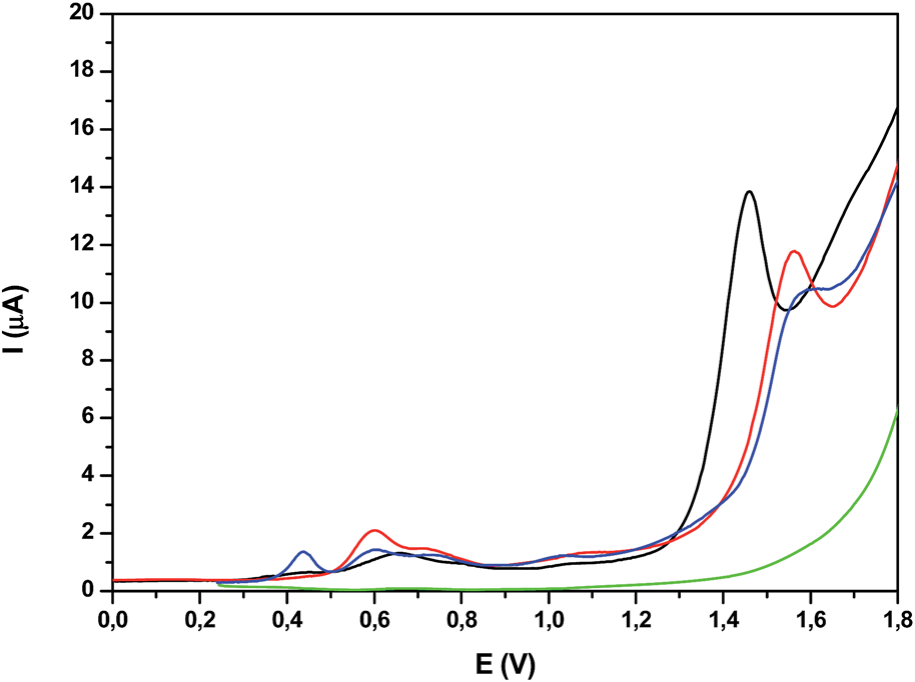
DPV of (black) **Ru_2_Co–(H_2_O)_4_**, (red) **Ru_2_Mn–(H_2_O)_4_**, (blue) ***in*-1** and (green) blank in a pH = 7.0 (50 mM) phosphate buffer solution and CF_3_CH_2_OH mixture (19 : 1).

The redox properties of the trinuclear complexes **Ru_2_Co–(H_2_O)_4_** and **Ru_2_Mn–(H_2_O)_4_** were investigated by means of CV and DPV in a mixture of a pH = 7.0 phosphate buffer (50 mM) and CF_3_CH_2_OH (19 : 1), the use of the latter forced by the poor solubility of the complexes in water. While very nicely defined waves were observed in organic solvents for their acetato counterparts as shown previously, in aqueous solution, the waves are very wide (Fig. S28†). Nevertheless, the DPVs in Fig. 6 allow the observation of three faradaic processes situated at approximately 0.6, 0.7 and 1.0 V for both **Ru_2_Co–(H_2_O)_4_** and **Ru_2_Mn–(H_2_O)_4_**, which are plotted together with the mononuclear aqua complex ***in*-1** for comparative purposes. However, the most important feature is the large electrocatalytic wave displayed by these complexes, starting at 1.3 V for **Ru_2_Co–(H_2_O)_4_** and at around 1.5 V for **Ru_2_Mn–(H_2_O)_4_** and ***in*-1**, which is associated to water oxidation catalysis.

### Water oxidation catalysis

Photochemically driven water oxidation catalysis has been carried out through the photogeneration of [Ru(bpy)_3_]^3+^ as a chemical oxidant using persulfate as a sacrificial electron acceptor (eqn (4)–(6)).

4 [Ru^II^(bpy)_3_]^2+^ + *hν* → [Ru^II^(bpy)_3_]^2+^*

5 [Ru^II^(bpy)_3_]^2+^* + S_2_O_8_^2–^ → [Ru^III^(bpy)_3_]^3+^ + SO_4_^2–^ + SO_4_^–^˙

6 [Ru^II^(bpy)_3_]^2+^ + SO_4_^–^˙ → [Ru^III^(bpy)_3_]^3+^ + SO_4_^2–^

**Fig. 7:**
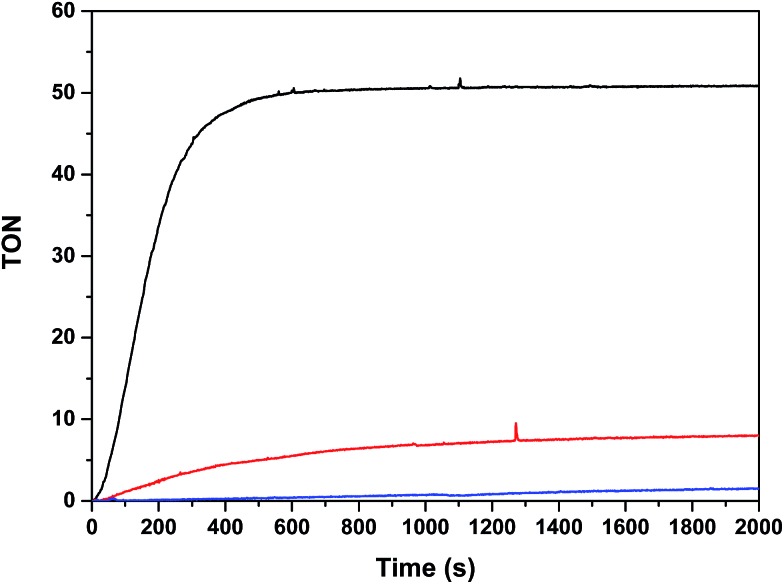
Photochemically induced oxidation of **Ru_2_Co–(H_2_O)_4_** (black), **Ru_2_Mn–(H_2_O)_4_** (red) and ***in*-1** (blue). Reaction conditions: [catalyst] = 50 μM, [[Ru(bpy)_3_](ClO_4_)_2_] = 0.5 mM; [Na_2_S_2_O_8_] = 20 mM; total volume = 2 mL in a pH = 7.0 (50 mM) phosphate buffer solution and CF_3_CH_2_OH mixture (19 : 1). A 300 W xenon lamp was used to illuminate the sample through a band pass filter of 440 nm at 298 K. The O_2_ yields based on persulfate are: 25%, 4% and <1%, respectively, for **Ru_2_Co–(H_2_O)_4_**, **Ru_2_Mn–(H_2_O)_4_** and ***in*-1**.

The oxygen generation profile as a function of time is presented in Fig. 7 for the systems Cat 50 μM/[Ru(bpy)_3_]^2+^ 0.5 mM/Na_2_S_2_O_8_ 20 mM/pH = 7.0 (50 mM phosphate buffer), with a total volume of 2.0 mL (H_2_O : CF_3_CH_2_OH = 19 : 1) using a 300 W xenon lamp with a band pass filter of 440 nm, thermostated at 298 K. Under these conditions, **Ru_2_Co–(H_2_O)_4_** generates a TON of 50 in about 10 minutes (TOF_i_ = 0.21 s^–1^) whereas **Ru_2_Mn–(H_2_O)_4_** only generates a TON of 8 and the mononuclear complex ***in*-1** does not generate any molecular oxygen within this time frame. These results are consistent with the electrochemical analysis, as the potentials for catalysis for **Ru_2_Mn–(H_2_O)_4_** and ***in*-1** are too high for efficient catalysis, given the fact that the Ru(iii)/Ru(ii) potential for [Ru(bpy)_3_]^2+/3+^ is *E*_1/2_ = 1.26 V.

**Fig. 8:**
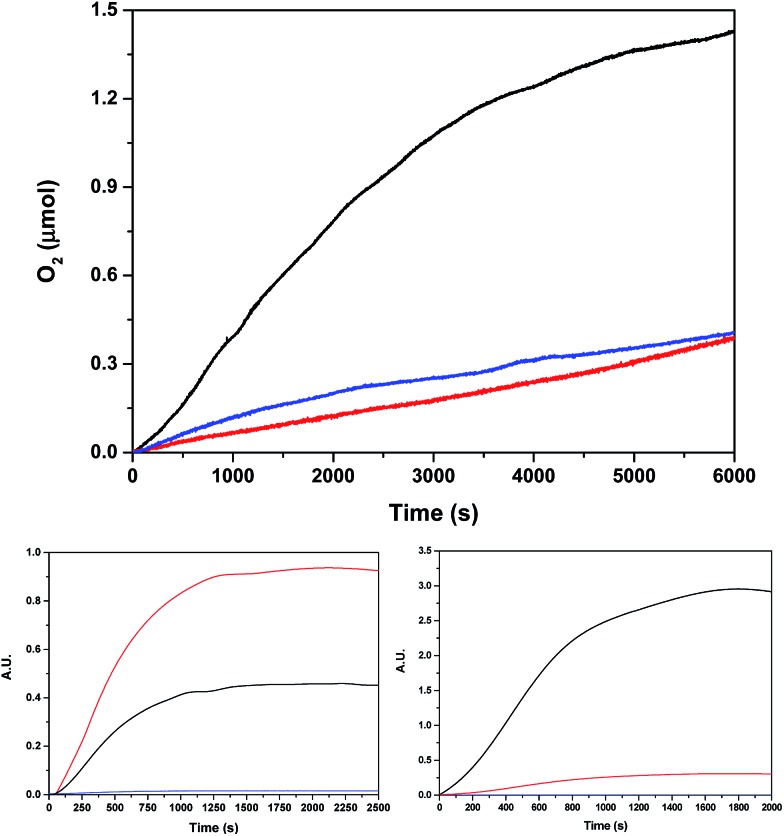
(Top) Oxygen evolution profile using oxone (4.7 mM) as a chemical oxidant for 40 μM **Ru_2_Co–(H_2_O)_4_** (black), 80 μM ***in*-1** (blue) and a blank without catalyst (red), using a pH = 7.0 (50 mM) phosphate buffer up to a total volume of 2.0 mL (H_2_O : CF_3_CH_2_OH = 19 : 1) in a 298 K thermostated cell. The O_2_ yields based on oxone for **Ru_2_Co–(H_2_O)_4_** and ***in*-1** are: 21% and <1%, respectively. (Bottom) Isotopic oxygen generation profile monitored by on-line MS for **Ru_2_Co–(H_2_O)_4_** using oxone in the same conditions. Bottom left using 97% H_2_^18^O, and bottom right using 15% H_2_^18^O. Color code: black ^32^O_2_, red ^34^O_2_, blue^36^O_2_.

In light of the good results obtained in the photoactivated experiment, water oxidation catalysis with **Ru_2_Co–(H_2_O)_4_**, and with the mononuclear ***in*-1** for comparison, was also investigated using oxone (KHSO_5_) as a chemical oxidant. The oxygen evolution profiles as a function of time are presented in Fig. 8. The Co containing complex generates 1.0 μmol of oxygen (0.41 μmol are subtracted due to the activity of the blank under the same conditions) that correspond to a TON of about 13.

Labelling experiments using H_2_^18^O were also carried out in order to extract mechanistic information regarding the O–O bond formation event when using oxone as a chemical oxidant. The O_2_ 36/34/32 isotope ratio was followed by on-line Mass spectrometry. Two different degrees of H_2_O labelling were employed, and the results obtained are shown in Fig. 8.

For discussion of the mechanism, we consider first the experiment with 97% H_2_^18^O, and then test our hypothesis on the data obtained from the experiment with 15% H_2_^18^O. However, before making a hypothesis on the mechanism of oxygen evolution, it is paramount to obtain information on the labeling state of the system at the moment of the oxygen evolving event.

(a). Raman experiments carried out under the same conditions as the water oxidation labelling experiments showed that no ^18^O exchange occurs at all between water and oxone for at least 10 h (Fig. S30†).^30^

(b). Substitution reactions of aqua ligands at low oxidation states (II) for Ru and Co complexes occur rapidly. As the precatalyst presents all acetate bridge ligands, we can assume that all the aqua ligands are initially present as H_2_^18^O.

(c). Peroxide oxidations of Ru-aqua polypyridyl complexes occur through dehydrogenation pathways.^31^ Thus, oxone will react with low oxidation states of Ru-aqua as follows (auxiliary ligands not shown):

7 Ru^II^–^18^OH_2_ + [HO–S(O)_2_(OO)]^–^ → Ru^IV^

<svg xmlns="http://www.w3.org/2000/svg" version="1.0" width="16.000000pt" height="16.000000pt" viewBox="0 0 16.000000 16.000000" preserveAspectRatio="xMidYMid meet"><metadata>
Created by potrace 1.16, written by Peter Selinger 2001-2019
</metadata><g transform="translate(1.000000,15.000000) scale(0.005147,-0.005147)" fill="currentColor" stroke="none"><path d="M0 1440 l0 -80 1360 0 1360 0 0 80 0 80 -1360 0 -1360 0 0 -80z M0 960 l0 -80 1360 0 1360 0 0 80 0 80 -1360 0 -1360 0 0 -80z"/></g></svg>


^18^O + H_2_O + HSO_4_^–^

(d). Oxidation of first row transition metals, particularly iron and cobalt polyridylic complexes, with peroxides occurs primarily *via* nucleophilic substitution.^32^–^34^

8 Co^II^–^18^OH_2_ + [HO–S(O)_2_(OO)]^–^ → Co^II^–OO–S(O)_2_(OH) + H_2_O^18^

9 Co^II^–OO–S(O)_2_(OH) → Co^IV^=O + HSO_4_^–^

(e). Tautomeric equilibrium between the oxo-hydroxo complexes, as described in eqn (10), would produce labelling scrambling, as has been earlier proposed for related complexes.^35^

10 Co^IV^(^18^O)(OH) ⇌ Co^IV^(O)(^18^OH)

**Scheme 2:**
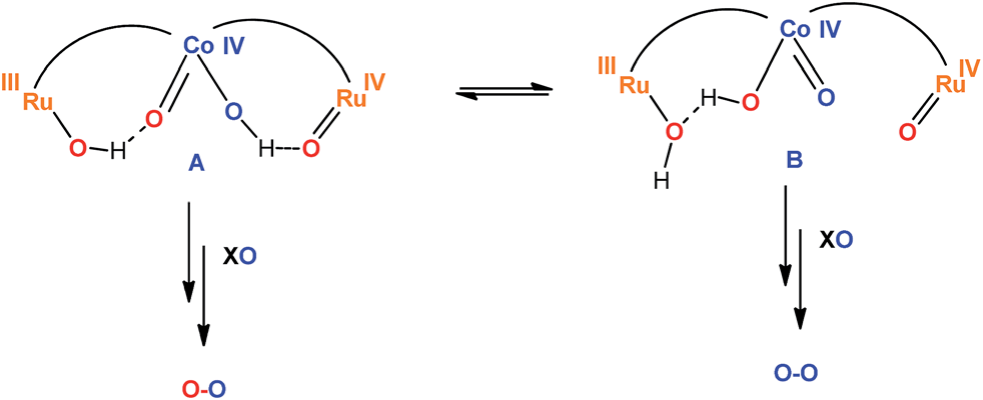
Fast tautomeric equilibrium proposed for the active species of the trinuclear **Ru_2_Co–(H_2_O)_4_** complex just before the O–O bond formation step. The bpp-ligand is represented by an arc and the trpy ligands are omitted for clarity purposes. XO represents the oxidant oxone (KHSO_5_). The blue oxygens are ^16^O and the red oxygens are ^18^O.

For the **Ru_2_Co–(H_2_O)_4_** complex, this situation at the oxygen evolving event is described in the top section of Scheme 2. This assumes that for 100% H_2_^18^O labeling at oxidation state II, all the aqua groups are exchanged. For the Ru center reaching high oxidation states with non-labeled oxone, [Ru(iv)= ^18^O] will be produced with 100% ^18^O labeling. However, for the cobalt center, a mixed labeled [Co(iv)(^16^O)(^18^OH)] will be generated because of the nucleophilic attack mechanism mentioned above.

At this point, the following conclusions are extrapolated from the oxygen data shown in Fig. 8.

(1). The practically negligible amount of ^36^O_2_ in the 97% H_2_^18^O experiment indicates the inexistence of intramolecular pathways between the Ru=O and Co=O moieties. In addition, it also precludes the existence of bimolecular pathways involving only the Ru=O groups. On the other hand, potential bimolecular pathways involving only Co centers are sterically highly disfavorable.

(2). The mononuclear complex ***in*-1** does not generate sufficient oxygen to be significant under the applied conditions and thus the inexistence of O_2_ coming independently from Ru-aqua/Ru-oxo moieties is ruled out.

(3). *Cis* O–O coupling within the same Co metal center would be compatible with the ratios of isotopic labelling obtained, although this mechanism has been discarded based on DFT in a number of examples.^36^,^37^

(4). The 97% labelling experiment (Fig. 8 and S31†) is consistent with the existence of a very fast tautomeric equilibrium where species A and B exist in a 2 : 1 ratio respectively (see Scheme 2). The origin of the higher stabilization of species A is proposed to come from the higher hydrogen bond capacity of isomer A with regard to that of B. Oxidation states of Ru and Co are tentatively assigned from the apparent removal of 4/5 electrons from the oxidation state II,II,II, as judged from the electrocatalytic wave displayed by the complex. Assuming this equilibrium, the reaction of oxone (XO) with A and B would generate molecular oxygen with a ratio of isotopes of ^34^O_2_/^32^O_2_ = 2/1, which is what is found experimentally.

(5). Finally, changing the ratio of labelled water to 15% ^18^O (Fig. 8 and S31†), will generate molecular oxygen with a ratio of ^34^O_2_/^32^O_2_ = 1/9. Experimentally, we obtained a ratio of 1/10, which is in very good agreement with the proposed mechanism.

Overall, the labelling experiments carried out together with the rest of the electrochemical and spectroscopic properties are in agreement with the presence of a water nucleophilic attack (WNA) mechanism occurring at the Co site of the trinuclear complex, with cooperative interaction of the two Ru sites *via* electronic coupling through the bpp^–^ bridging ligand and *via* neighboring hydrogen bonding.

It is worth mentioning that in our previous work with related dinuclear Ru^21^ and Co^3^ complexes containing the Hbpp ligand, namely {[M(H_2_O)(trpy)]_2_μ-[(bpp)}^3+^ (M = Ru or Co), the O–O bond formation occurred *via* an intramolecular mechanism (I2M).^38^ However, with the new trinuclear complexes described here, given the new substantially different geometries and electronic interactions between the metal centers, the mechanism changes to a WNA.

## Conclusions

We have prepared and isolated a family of trinuclear complexes, Ru_2_M–X_2_ (M=Co or Mn; X = Cl^–^ or OAc^–^), where the central Co or Mn atom, together with two bpp^–^ ligands, acts as a bridge between the two external Ru moieties. These complexes have been characterized in the solid state by X-ray diffraction analysis and by magnetic measurements. In solution they have been characterized by spectroscopic (EPR, UV-vis) and by electrochemical (CV, DPV) techniques. Overall, all these experiments for the chlorido or acetato bridge complexes show the presence of a relatively weak electronic coupling between the metal centers, transmitted through the bridging ligands. In addition, it is very interesting to see how the redox pattern radically changes from the Mn complexes with regards to the Co complexes manifesting the intrinsically different electronic properties of the two transition metals. In aqueous acidic media the trinuclear complexes revert to their Ru mononuclear counterparts and free Co(ii) or Mn(ii). However at pH = 7.0 the integrity of the complex is fully retained. Of particularly interest is the heterotrinuclear **Ru_2_Co–(H_2_O)_4_** complex, that is capable at this pH of catalytically oxidizing water to molecular dioxygen chemically, electrochemically and photochemically. Finally, while a few detailed H_2_^18^O labelling experiments have been described for Ru complexes^13^,^39^–^51^ that allow the tracing of the O–O bond formation step, this type of information is lacking for first row transition water oxidation catalysts. Our H_2_^18^O labelling experiments constitute the first example where the O–O bond formation step has been elucidated based on labeling experiments.

## Supplementary Material

Supplementary information

Crystal structure data
