# Supplementary material for: Synthesis, structure, spectroscopy and reactivity of new heterotrinuclear water oxidation catalysts
Source: Chem Sci. 2016 Feb 2;7(5):3304–12. doi: 10.1039/c5sc04672f (PMC6006862; doi:10.1039/c5sc04672f)

Electronic Supplementary Material (ESI) for Chemical Science.
This journal is © The Royal Society of Chemistry 2016

Supporting Info for

Synthesis, Structure, Spectroscopy and Reactivity of New Heterotrinuclear Water Oxidation Catalysts

Lorenzo Mognon,[a] Sukanta Mandal,[b] Carmen E. Castillo,[c,d] Jérôme Fortage,[c,d] Florian Molton,[c,d] Guillem Aromi,[e] Jordi Benet-Buchhlolz,[a] Marie-Noëlle Collomb,[c,d] and Antoni Llobet[a,f,*]

[a] Institute of Chemical Research of Catalonia (ICIQ), Barcelona Institute of Science and Technology, Avinguda Països Catalans 16, 43007 Tarragona, Spain

[b] Department of Chemistry, Indian Institute of Technology Kharagpur,Kharagpur-721302, West Bengal, India

[c] Univ. Grenoble Alpes, DCM, F-38000 Grenoble, France

[d] CNRS, DCM, F-38000 Grenoble, France

[e] Departament de Química Inorgànica, Universitat de Barcelona, Diagonal 645, 08028 Barcelona, Spain

[f] Departament de Química, Universitat Autònoma de Barcelona, Cerdanyola del Vallès, 08193 Barcelona, Spain

Experimental Section

Preparations.

All reagents used in the present work were obtained from Aldrich Chemical Co. and were used without further purification. Solvents were purchased from SDS, and they were purified and dried either by passing them through an activated alumina purification system (MBraun SPS-800) or by conventional distillation techniques. *Out*-[Ru(Cl)(Hbpp)(trpy)][PF_6], ***out*-0**, and [Ru(bpy)$_3$][ClO_4]$_2$ were synthesized as reported in literature.[1]

{[Ru^{II}(trpy)]$_2$(μ-[Co^{II}(Cl)$_2$(bpp)$_2$])}(PF_6)$_2$, Ru$_2$Co-Cl$_2$.

Out-[Ru(Cl)(Hbpp)(trpy)] [PF_6] (100 mg, 0.136 mmol) was placed in a 250 mL round-bottom flask and dissolved in 200 mL of methanol. A sample of NaOMe (8 mg, 0.142 mmol) was added and the solution stirred for 30 minutes. The reaction was then kept under 200 W light irradiation (tungsten lamp) overnight and after that 5 mL methanol/water (4:1) solution of $CoCl_2$ (25 mg, 0.190 mmol) and LiCl (57 mg, 1.344 mmol) were added. The reaction mixture was heated at 60 °C under 200 W tungsten lamp light for 5 h. It was then filtered and the volume was reduced to around 20 mL under reduced pressure. Saturated aqueous solution of KPF_6 (2 mL) was added, giving rise to a reddish-brown precipitate. The mixture was then cooled in an ice bath for 30 minutes and the solid filtered on a frit, washed with cold water (3 times) and then with diethyl ether (3 times), and finally dried under vacuum. Yield: 60 mg (57.6 %). Anal. Calcd for $C_{56}H_{40}Cl_2CoF_{12}N_{14}P_2Ru_2 \cdot 2H_2O$: C 42.92, H 2.83, N 12.51. Found: C 42.98, H 2.88, N 12.46. MALDI (DCTB matrix, CH_2Cl_2): 1386.0 ([M-PF_6]$^+$). UV-vis [λ_{max}, nm (ε, M^{-1} cm^{-1})]: (in CH_3CN) 510 (14470), 492 (14620), 408 (sh) (20380), 372 (25650), 315 (78330), 275 (74310), 234 (73100); (in CH_2Cl_2) 518 (sh)(12900), 495 (13340), 410 (sh) (18640), 375 (23060), 316 (68430), 276 (64500). IR (KBr, cm^{-1}, selected bands): 843 (ν(PF_6^-)).

{[Ru^{II}(trpy)]$_2$(μ-[Mn^{II}(Cl)$_2$(bpp)$_2$])}(PF_6)$_2$, Ru$_2$Mn-Cl$_2$.

Out-[Ru(Cl)(Hbpp)(trpy)] [PF_6] (100 mg, 0.136 mmol) was placed in a 250 ml round-bottom flask and dissolved in 200 mL of methanol. A sample of NaOMe (15 mg, 0.278 mmol) was added and the solution stirred for 30 minutes. A solution of $MnCl_2 \cdot 4H_2O$ (27 mg, 0.136 mmol) and LiCl (57 mg, 1.344 mmol) in 5 mL of methanol/water (4:1) was added and the reaction mixture was heated at 60 °C for 4 hours. The solution was allowed to cool down and then stirred overnight under 200 W light irradiation (tungsten lamp) at room temperature. The resulting mixture was kept in fridge (5 °C) for 1 h and was then filtered. The volume was reduced to around 20 mL under reduced pressure. Saturated aqueous solution of KPF_6 (2 mL) was added, giving rise to a reddish-brown precipitate. The reaction mixture was then cooled in an ice bath for 30 minutes and the solid filtered on a frit, washed with cold water (3 times) and then with diethyl ether (3 times), and finally dried under vacuum. Yield: 73 mg (70.2 %). Anal. Calcd for $C_{56}H_{40}Cl_2F_{12}MnN_{14}P_2Ru_2 \cdot 2H_2O$: C 43.03, H 2.84, N 12.55. Found: C 43.13, H 2.72, N 12.15. MALDI (DCTB matrix, CH_2Cl_2): 1382.1 ([M-PF_6]$^+$). UV-vis [λ_{max}, nm (ε, M^{-1} cm^{-1})]: (in CH_3CN) 515 (17026), 485 (18140), 408 (sh) (22830), 368 (30273), 315 (76130), 302 (78110), 276 (79180), 234 (73525); (in CH_2Cl_2) 520 (sh)(17380), 490 (19125), 410 (sh) (23330), 370 (31456), 317 (78200), 302 (84190), 282 (sh) (79700), 277 (81925). IR (KBr, cm^{-1}, selected bands): 843 (ν(PF_6^-)).

General Synthetic Procedure for Ru$_2$Co-OAc$_2$ and Ru$_2$Mn-OAc$_2$.

A sample of **Ru$_2$Co-Cl$_2$** (50 mg, 0.033 mmol) or **Ru$_2$Mn-Cl$_2$** (50 mg, 0.033 mmol), respectively, and NaOAc (0.030 g 0.365 mmol) was dissolved in 15 mL of an acetone/water (5:1) mixture and heated at 75°C for 3 h. After cooling at room temperature the resulting solution was filtered and few drops of saturated aqueous solution of KPF_6 were added. Upon reduction of the volume, a solid came out from the solution that was filtered and washed with cold water (3 times) and then with diethyl ether and finally dried under vacuum. The yields and characterizations of the complexes are given below:

{[Ru^{II}(trpy)]$_2$(μ-[Co^{II}(AcO)$_2$(bpp)$_2$])}(PF_6)$_2$, Ru$_2$Co-OAc$_2$.

Yield: 0.040 g (73.4%). Anal. Calcd for $C_{60}H_{46}CoF_{12}N_{14}O_4P_2Ru_2$: C 45.67, H 2.94, N 12.43. Found: C 45.62, H 2.82, N 12.56. ESI-MS (in MeOH): 1434.1 ([M-PF_6]$^+$); 644.5 ([M-2PF_6]$^{2+}$). UV-vis [λ_{max}, nm (ε, M^{-1} cm^{-1})]: (in CH_3CN) 540 (sh) (12697), 506 (15307), 412 (sh) (16614), 368 (26614), 314 (82077), 276 (75931), 234 (67057); (in CH_2Cl_2) 540 (sh) (13587), 506 (16103), 412 (sh) (17771), 368 (27627), 314 (81785), 276 (75910), 236 (68229). IR (KBr, cm^{-1}, selected bands): 1604, 1568 (ν_{asym}(O_2CMe)); 1447, 1437, 1416 (ν_{sym}(O_2CMe)); 843 (ν(PF_6^-)).

{[Ru^{II}(trpy)]$_2$(μ-[Mn^{II}(AcO)$_2$(bpp)$_2$])}(PF_6)$_2$, Ru$_2$Mn-OAc$_2$.

Yield: 0.040 g (73.6%). Anal. Calcd for $C_{60}H_{46}F_{12}MnN_{14}O_4P_2Ru_2$: C 45.78, H 2.95, N 12.46. Found: C 45.90, H 2.99, N 12.51. ESI-MS (in MeOH): 1430.1 ([M-PF_6]$^+$); 642.5 ([M-2PF_6]$^{2+}$). UV-vis [λ_{max}, nm (ε, M^{-1} cm^{-1})]: (in CH_3CN) 538 (sh) (12815), 504(15384), 410 (sh) (17070), 365 (26520), 318 (71463), 306 (71388), 278 (68937), 234 (62473); (in CH_2Cl_2) 538 (sh) (13620), 504 (16069), 410 (sh) (17862), 365 (27603), 318 (71381), 306 (72999), 278 (70850), 234 (64346). IR (KBr, cm^{-1}, selected bands): 1601, 1568 (ν_{asym}(O_2CMe)); 1446, 1436, 1421 (ν_{sym}(O_2CMe)); 843 (ν(PF_6^-)).

Instruments and measurements.

Physical methods.

Elemental analysis were performed using a CHNS-O EA-1108 elemental analyser from Fisons. IR spectra of solid samples were taken in Bruker Vector 22 FT-IR spectrophotometer (in KBr, 4000−500 cm^{-1}). UV-vis spectra were recorded either in Agilent 8453 diode-array spectrophotometer or Cary 50 scan spectrometer (experimental errors on the measurement for extinction coefficients are less than 1%). This is now indicated in the experimental section. The cell path length was 1 mm. MS analyses were recorded on an esquire 6000 ESI ion trap LC/MS (Bruker Daltonics) equipped with an electrospray ion source and MALDI were recorded in Bruker Autoflex.

Electrochemistry.

The electrochemical measurements were run under an argon atmosphere at room temperature. When CH_3CN was used as solvent, the electrochemical measurements were performed in a dry-glove box. Cyclic voltammetry experiments were performed using an EG&G model 173 potentiostat/galvanostat equipped with a PAR model universal programmer and a PAR model 179 digital coulometer. A standard three-electrode electrochemical cell was used. Potentials were measured with an Ag/0.01 M $AgNO_3$ reference electrode in a solution 0.1 M [($n$$Bu_4N$)$ClO_4$] in CH_3CN, or with an aqueous Saturated Calomel Electrode (SCE) reference electrode in a pH = 7.0 phosphate buffer solution and CF_3CH_2OH mixture (19:1). Potentials referred to Ag/$AgNO_3$ system can be converted to the normal hydrogen electrode (NHE) by adding 548 mV. Potentials referred to SCE system can be converted to NHE electrode by adding 205 mV. The working electrodes, polished with 2 µm diamond paste (Mecaprex Presi), were a platinum disk (5 mm in diameter) or a carbon vitreous disk (3 mm in diameter) for cyclic voltammetry. For rotating disk electrode (RDE) experiments, a carbon disk (2 mm in diameter) was used. The auxiliary electrode was a Pt wire. All the potentials are reported against NHE, unless otherwise indicated.
For experiments performed in glove box, progress of electrolysis was followed by the change in UV-Vis spectra with a MCS 501 UV-NIR (Carl Zeiss) spectrophotometer equipped with an automatic shutter. The light sources are halogen (CLH 500 20 W) and deuterium lamps (CLD 500) with optic fibers (041.002–UV SN 012105). For experiments performed in water, UV-visible spectra were recorded on a Variant Cary 300. The cell path length was 1 mm.

EPR spectroscopy.

X-band EPR spectra were recorded with a Bruker EMX, equipped with the ER-4192 ST Bruker cavity. The simulations were performed using the Bruker XSophe software (version 1.1.4.).

Magnetic susceptibility.

The variable-temperature magnetic susceptibility data of crystalline samples of **Ru_2Mn-Cl_2**, **Ru_2Mn-OAc_2**, **Ru_2Co-Cl_2** and **Ru_2Co-OAc_2** were collected with Quantum Design MPMS5 SQUID magnetometer at the Centres Científics i Tecnològics (CCiT) of the Universitat de Barcelona. Pascal's constants were used to estimate diamagnetic corrections to the molar paramagnetic susceptibility, and a correction was applied for the sample holder. For sample **Ru_2Mn-Cl_2** the data were also corrected for a very residual amount of Ru^0 impurity, which introduced a slope to the χT *vs* T curve.

Photochemical oxidations.

The photochemical oxygen evolution was carried out under irradiation with 300 W xenon lamp (MAX 302) with band pass filter 440 nm in a custom-made glass vessel with a water jacket. The temperature of the cell was maintained at 298 K by continuous flow of water connected with a thermostat. Oxygen evolution was analysed with a gas phase Clark type oxygen electrode (Unisense Ox-N needle microsensor). Two-point calibration was performed under air saturated and nitrogen saturated conditions. The photochemical experiment involved a three component system: catalyst, sensitizer ($[Ru(bpy)_3]^{2+}$) and sacrificial electron acceptor ($Na_2S_2O_8$). The pH was maintained at 7.04 with 50 mM phosphate buffer (no salt was added to adjust the ionic strength).Three stock solutions were prepared: catalyst (1 mM in CF_3CH_2OH), $[Ru(bpy)_3](ClO_4)_2$ (1 mM in pH = 7.04 aqueous phosphate buffer) and $Na_2S_2O_8$ (44.5 mM in pH = 7.04 aqueous phosphate buffer). 1 mL $[Ru(bpy)_3](ClO_4)_2$ and 0.9 mL $Na_2S_2O_8$ from the stock were taken together in the custom-made glass vessel and then 0.1 mL stock solution of catalyst was added under stirring conditions {$[catalyst]_f$ = 50 µM, $[[Ru(bpy)_3](ClO_4)_2]_f$ = 0.5 mM; $[Na_2S_2O_8]_f$ = 20 mM; total volume = 2 mL}. The glass vessel was sealed with rubber septum and the oxygen needle sensor was inserted through the rubber septum. The solution was thoroughly degassed and then the catalysis was initiated with irradiation of light.

Chemical water oxidation.

Chemical water oxidation catalysis was performed in a jacketed vessel thermostated at 298 K. A CF_3CH_2OH solution of the catalyst was introduced in the vessel and thoroughly deaerated. Previously degassed phosphate buffer was introduced under nitrogen atmosphere and stirring. Finally a solution of oxidant was added. The headspace gaseous content was monitored with a gas-phase Clark electrode, and successively calibrated with known additions of air. On-line mass

measurement of the gas evolution for labelling experiments was performed on a Pfeiffer Omnistar GSD 301C mass spectrometer, in the same conditions as the Chemical water oxidation.

X-ray Crystal Structure Determination.

Crystals of **Ru_2Mn-Cl_2** were obtained by slow diffusion of diethyl ether into an acetonitrile solution of the complex. Crystals of **Ru_2Mn-OAc_2** were obtained by slow evaporation of a methanol solution of the complex. The measured crystals were prepared under inert conditions immersed in perfluoropolyether as protecting oil for manipulation.

Data collection: Crystal structure determination for **Ru_2Mn-Cl_2** and **Ru_2Mn-OAc_2** was carried out using a Bruker-Nonius diffractometer equipped with an APPEX 2 4K CCD area detector, a FR591 rotating anode with MoK_α radiation, Montel mirrors as monochromator and an Oxford Cryosystems low temperature device Cryostream 700 plus (T = -173 $^{\circ}$C). Full-sphere data collection was used with ω and φ scans. Programs used: Data collection APEX-2,[2] data reduction Bruker Saint[3] V/.60A and absorption correction SADABS.[4,5]

Structure Solution and Refinement: Crystal structure solution was achieved using direct methods as implemented in SHELXTL[6] and visualized using the program XP. Missing atoms were subsequently located from difference Fourier synthesis and added to the atom list. Least-squares refinement on F^2 using all measured intensities was carried out using the program SHELXTL. All non hydrogen atoms were refined including anisotropic displacement parameters.

Comments to the structures: **Ru_2Mn-Cl_2**: The unit cell contains one molecule of the complex, two PF_6 anions, one diethyl ether molecule, one acetonitrile molecule and two water molecules. One of the water molecules is disordered in two positions (ratio: 56:44). **Ru_2Mn-OAc_2**: The unit cell contains one molecule of the complex, two PF_6 anions and one molecule of methanol. One of the PF_6 anions is disordered in two orientations. The methanol molecule is also disordered in two orientations (ratio 59:31). The measured sample was formed by two crystals with a ratio of 70:30. The collected data for both crystals were processed with TWINABS taking in account overlapping reflections.[7,5]. The measured crystals were of extreme small dimensions and although the measured sample was formed by two crystals only a completeness of 92 % was reached. Not better data could be collected.

Notes and references

1. C. Sens, M. Rodríguez, I. Romero, A. Llobet, T. Parella and J. Benet-Buchholz, *Inorg. Chem.*, 2003, **42**, 8385-8394.
2. Data collection: APEX II, versions v2009.1-02 and v2013.4-1; Bruker AXS Inc.: Madison, WI, 2007.
3. Data reduction: SAINT, versions V7.60A and V8.30c; Bruker AXS Inc.: Madison, WI, 2007.
4. SADABS, V2008/1 and V2012/1; Bruker AXS Inc.: Madison, WI, 2001.
5. R. Blessing, *Acta Crystallogr. Sect. A*, 1995, **51**, 33-38.
6. G. Sheldrick, *Acta Crystallogr. Sect. A*, 2008, **64**, 112-122.
7. TWINABS, version 2008/4 Bruker AXS Inc.: Madison, WI.

Table S1. Crystal data and structure refinement for $\mathbf{Ru_2Mn\text{-}Cl_2}$.

Empirical formula	C62 H57 Cl2 F12 Mn N15 O3 P2 Ru2
Formula weight	1678.15
Temperature	100(2) K
Wavelength	0.71073 Å
Crystal system	Triclinic
Space group	P-1
Unit cell dimensions	a = 14.6868(18) Å α= 69.889(3) °.
	b = 15.5818(14) Å β = 66.513(2) °.
	c = 17.997(2) Å γ = 67.869(2) °.
Volume	3406.8(7) $Å^3$
Z	2
Density (calculated)	1.636 Mg/m^3
Absorption coefficient	0.836 mm^{-1}
F(000)	1686
Crystal size	0.10 x 0.05 x 0.05 mm^3
Theta range for data collection	1.45 to 32.93 °.
Index ranges	-22<=h<=22 ,-23 <=k<=23 ,-27<=l<=27
Reflections collected	24640
Independent reflections	13360 [R(int) = 0.0934]
Completeness to theta = 32.93°	0.964 %
Absorption correction	Empirical
Max. and min. transmission	0.9594 and 0.9211
Refinement method	Full-matrix least-squares on F^2
Data / restraints / parameters	24640 / 12 / 905
Goodness-of-fit on F^2	1.019
Final R indices [I>2sigma(I)]	R1 = 0.0741 , wR2 = 0.1992
R indices (all data)	R1 = 0.1549 , wR2 = 0.2546
Largest diff. peak and hole	3.138 and -1.357 e.$Å^{-3}$

Table S2. Crystal data and structure refinement for **Ru_2Mn-OAc_2**.

Empirical formula	C61 H50 F12 Mn N14 O5 P2 Ru2
Formula weight	1606.17
Temperature	100(2) K
Wavelength	0.71073 Å
Crystal system	Triclinic
Space group	P-1
Unit cell dimensions	a = 14.471(3) Å α= 114.334(5) °.
	b = 15.501(4) Åβ = 112.431(4) °.
	c = 16.913(4) Åγ = 91.203(5) °.
Volume	3123.0(13) Å^3
Z	2
Density (calculated)	1.708 Mg/m^3
Absorption coefficient	0.827 mm^{-1}
F(000)	1610
Crystal size	0.10 x 0.10 x 0.01 mm^3
Theta range for data collection	2.36 to 30.66 °
Index ranges	-20 <=h<=18 ,-21 <=k<=20 ,0 <=l<=24
Reflections collected	17781
Independent reflections	17781 [R(int) = 0.0000]
Completeness to theta = 30.66°	92.0%
Absorption correction	Empirical
Max. and min. transmission	0.9918 and 0.9219
Refinement method	Full-matrix least-squares on F^2
Data / restraints / parameters	17781 / 32 / 937
Goodness-of-fit on F^2	1.036
Final R indices [I>2sigma(I)]	R1 = 0.0696 , wR2 = 0.1485
R indices (all data)	R1 = 0.1433 , wR2 = 0.1785
Largest diff. peak and hole	1.857 and -1.081 e.Å^{-3}

Figure S1. Mass spectra (MALDI+) of {[Ru^{II}(trpy)]$_2$(μ-[Co^{II}(Cl)$_2$(bpp)$_2$])}(PF$_6$)$_2$, **Ru$_2$Co-Cl$_2$**, in dctb-CH$_2$Cl$_2$. The peak at 1386.0 corresponds to cation [M-PF$_6$]$^+$; experimental (left) and simulated (right).

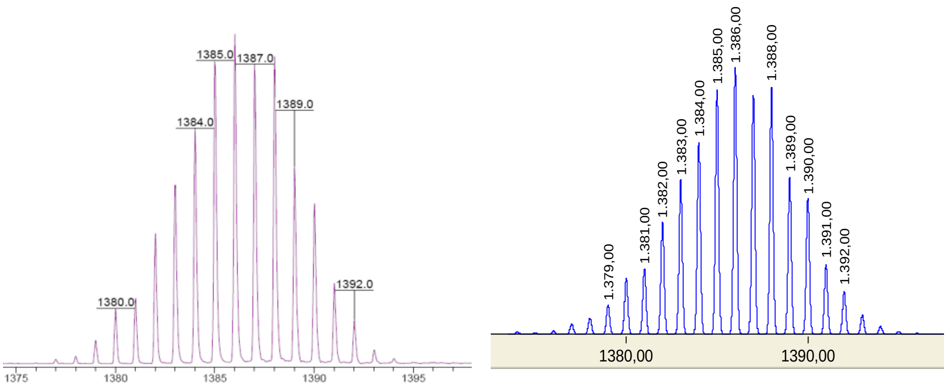

Figure S2. Mass spectra (MALDI+) of {[Ru^{II}(trpy)]$_2$(μ-[Mn^{II}(Cl)$_2$(bpp)$_2$])}(PF$_6$)$_2$, **Ru$_2$Mn-Cl$_2$**, in dctb-CH$_2$Cl$_2$. The peak at 1382.1 corresponds to cation [M-PF$_6$]$^+$; experimental (left) and simulated (right).

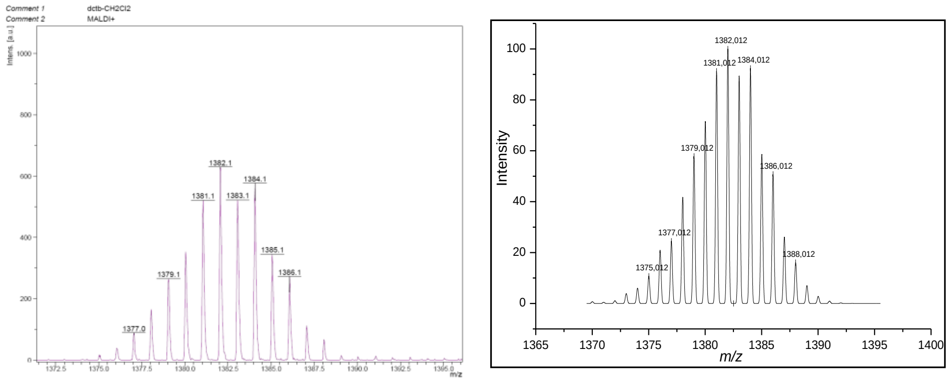

Figure S3. ESI Mass spectra of {[Ru^{II}(trpy)]$_2$(μ-[Co^{II}(AcO)$_2$(bpp)$_2$])}(PF_6)$_2$, **Ru_2Co-OAc_2**, in MeOH. (a) the peak at 1434 corresponds to cation [M-PF_6]+;Experimental (left) and simulated (right); the peak at 644.5 corresponds to cation [M-2PF_6]$^{2+}$;Experimental (left) and simulated (right).

(a)

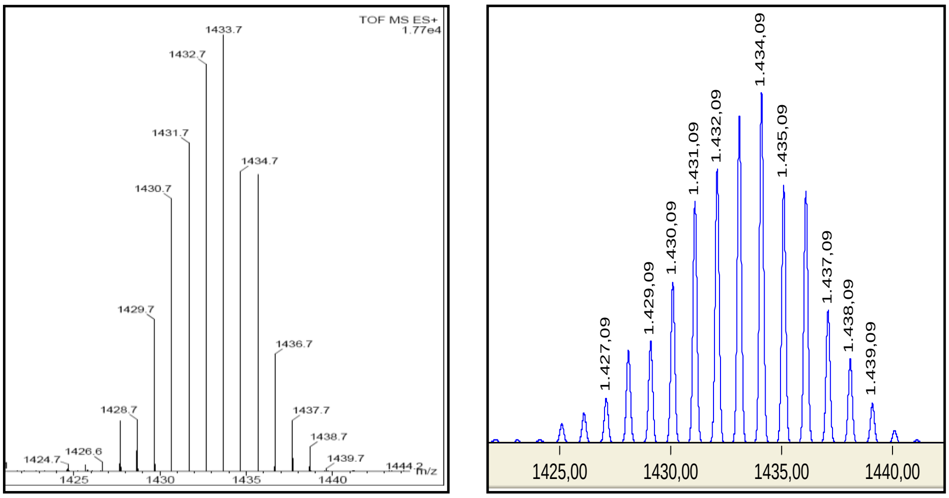

(b)

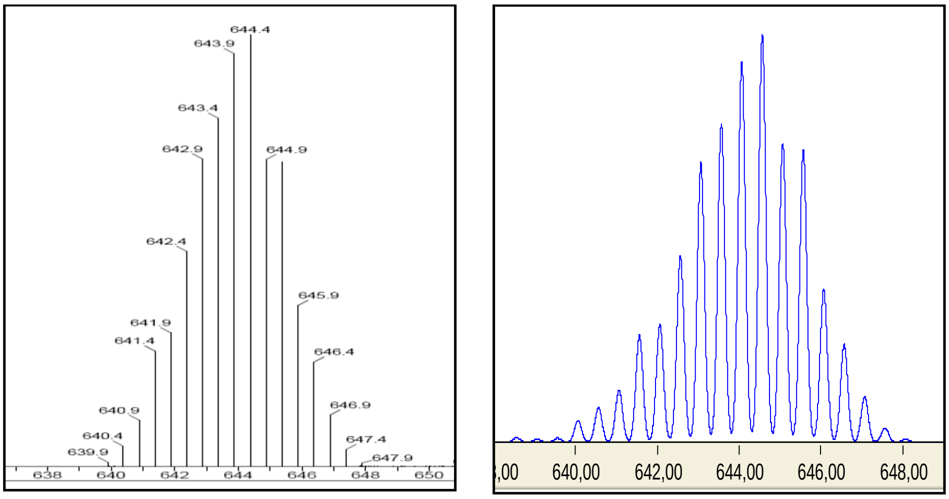

Figure S4. ESI Mass spectra of $\{[Ru^{II}(trpy)]_2(\mu\text{-}[Mn^{II}(AcO)_2(bpp)_2])\}(PF_6)_2$, **$Ru_2Mn\text{-}OAc_2$,** in MeOH. (a) The peak at 1430 corresponds to cation $[M\text{-}PF_6]^+$; Experimental (left) and simulated (right); (b) the peak at 642.5 corresponds to cation $[M\text{-}2PF_6]^{2+}$;Experimental (left) and simulated (right).

(a)

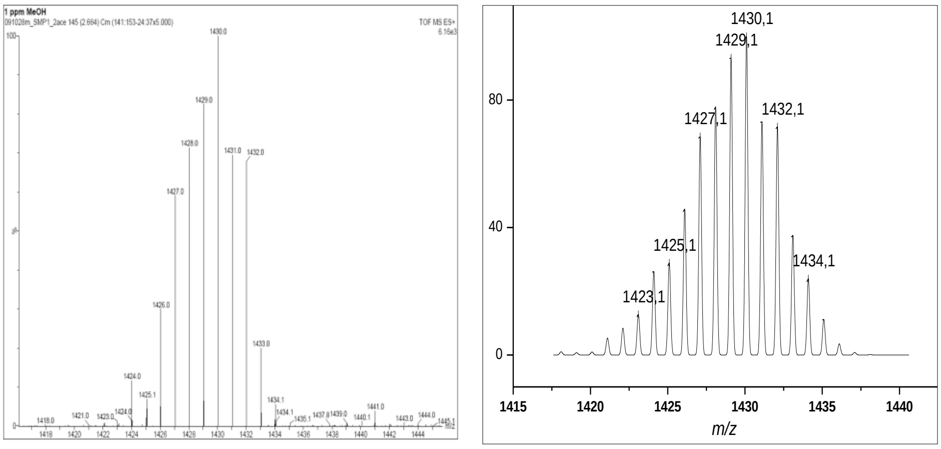

(b)

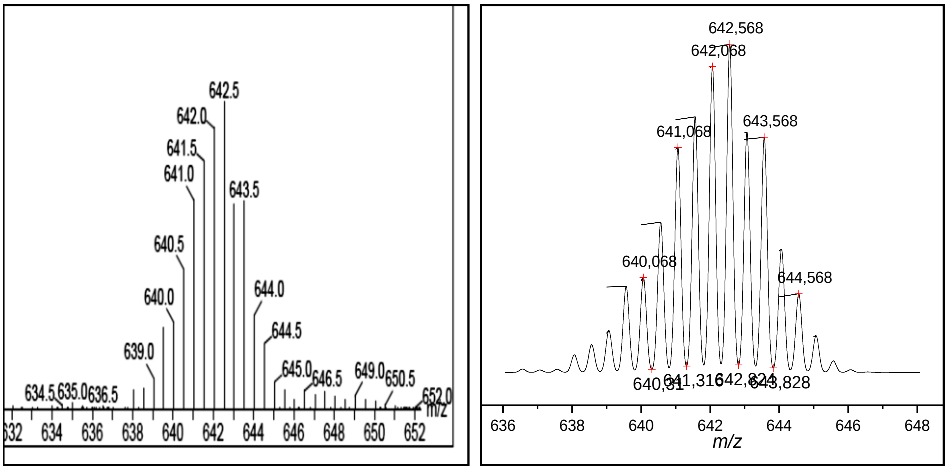

Figure S5. UV-vis spectra of {[Ru^{II}(trpy)]$_2$(μ-[Co^{II}(Cl)$_2$(bpp)$_2$])}(PF$_6$)$_2$, **Ru$_2$Co-Cl$_2$**, in (a) CH_3CN and (b) CH_2Cl_2.

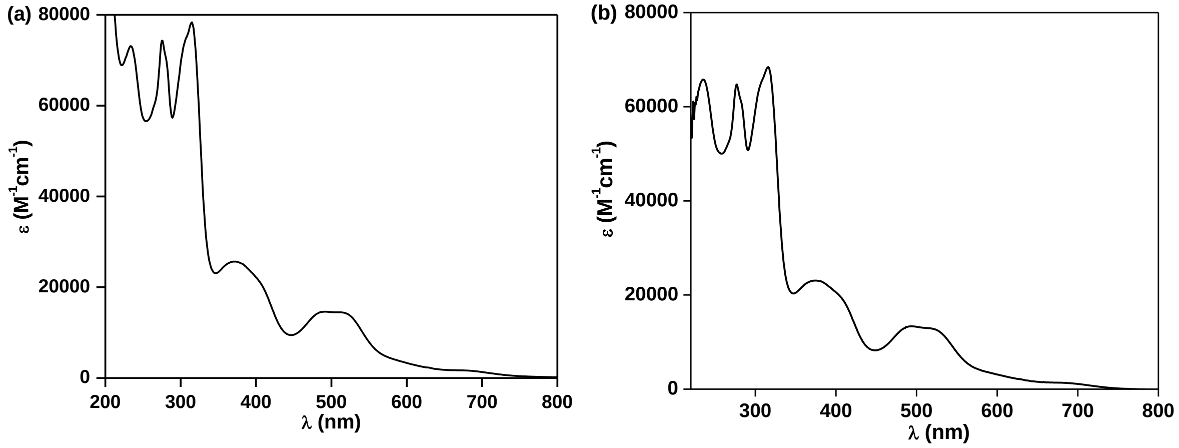

Figure S6. UV-vis spectra of {[Ru^{II}(trpy)]$_2$(μ-[Mn^{II}(Cl)$_2$(bpp)$_2$])}(PF$_6$)$_2$, **Ru$_2$Mn-Cl$_2$**, in (a) CH_3CN and (b) CH_2Cl_2.

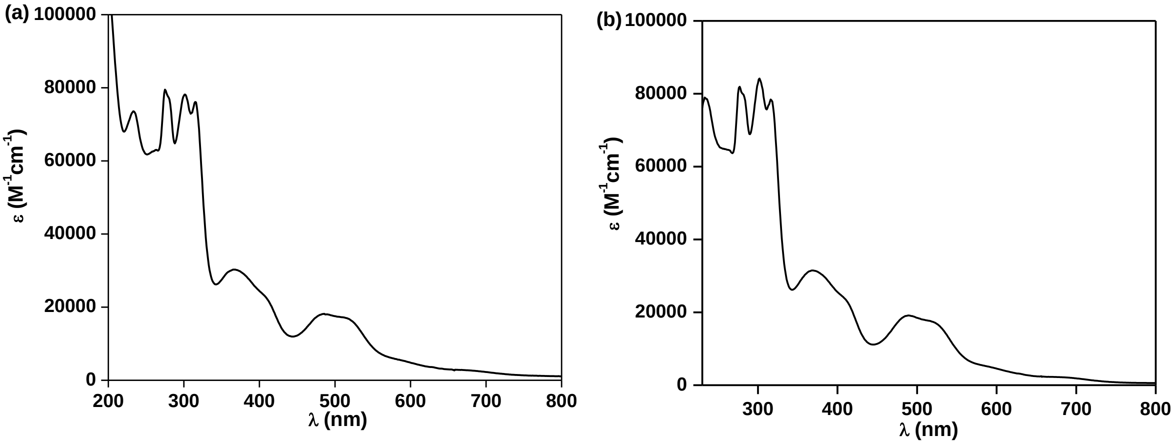

Figure S7. UV-vis spectra of {[Ru^{II}(trpy)]$_2$(μ-[Co^{II}(AcO)$_2$(bpp)$_2$])}(PF$_6$)$_2$, **Ru$_2$Co-OAc$_2$**, in (a) CH_3CN and (b) CH_2Cl_2.

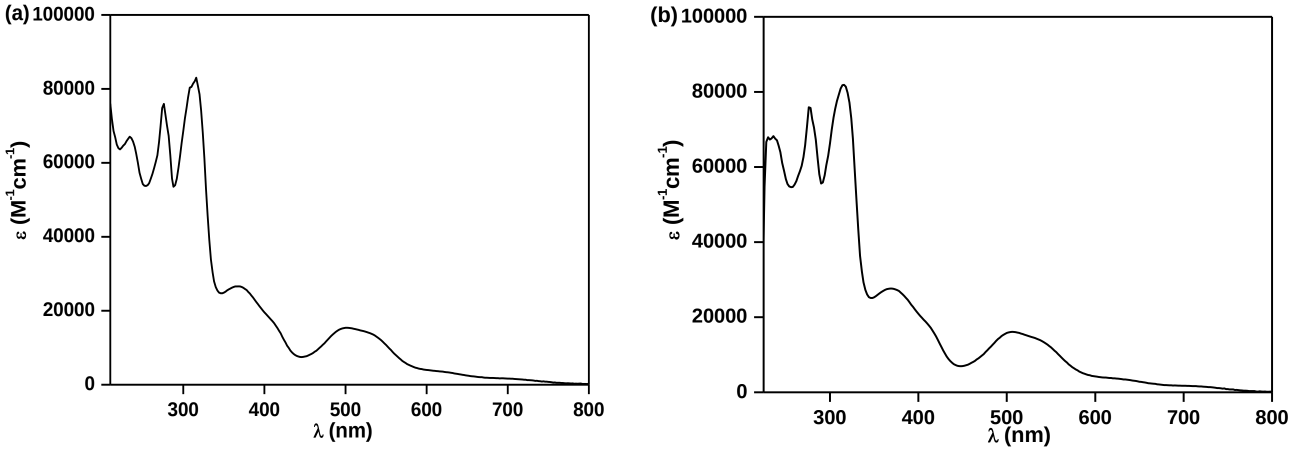

Figure S8. UV-vis spectra of {[Ru^{II}(trpy)]$_2$(μ-[Mn^{II}(AcO)$_2$(bpp)$_2$])}(PF$_6$)$_2$, **Ru$_2$CMn-OAc$_2$**, in (a) CH_3CN and (b) CH_2Cl_2.

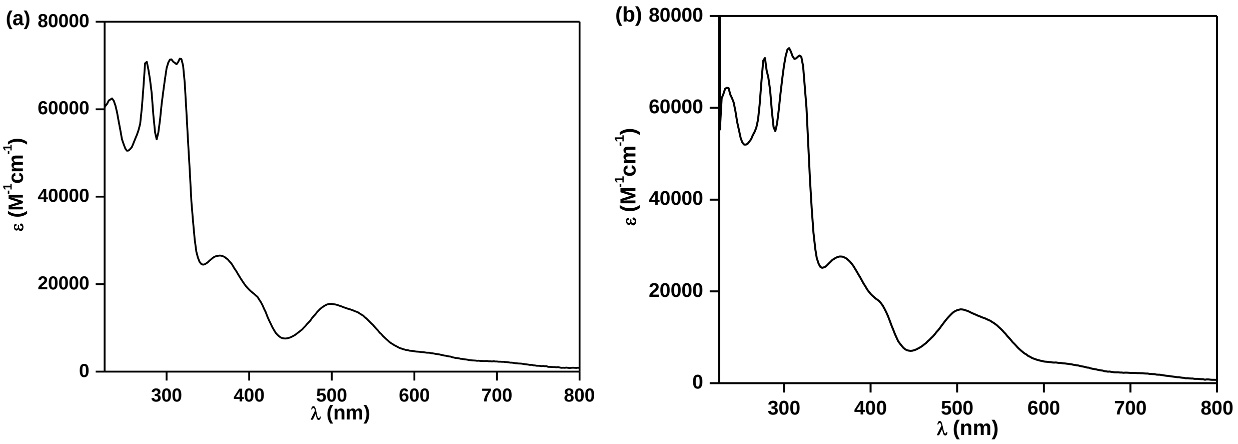

Figure S9. UV-visible absorption spectra of **Ru$_2$Co-OAc$_2$** (red) in CH_3CN and of **Ru$_2$Mn-OAc$_2$** (black) in CH_3CN.

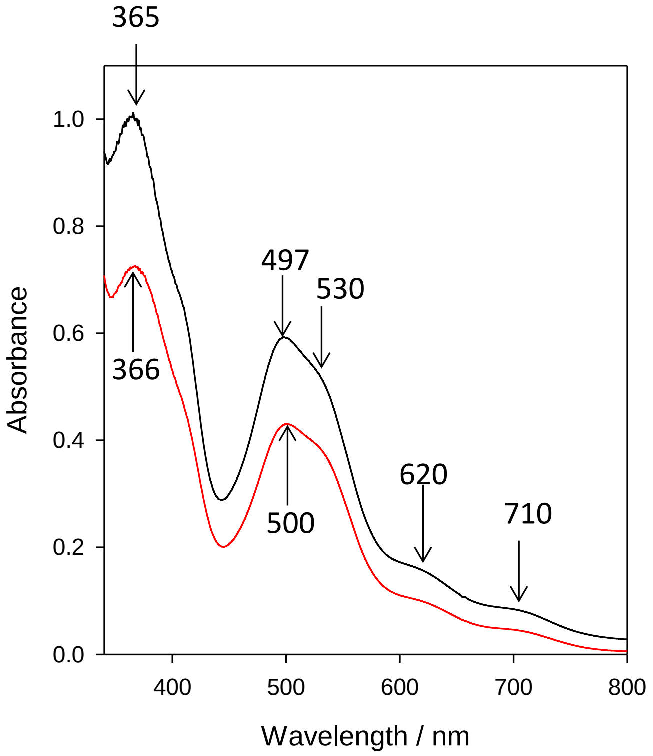

Figure S10. IR spectra (KBr) of {[Ru^{II}(trpy)]$_2$(μ-[Co^{II}(Cl)$_2$(bpp)$_2$])}(PF$_6$)$_2$, **Ru$_2$Co-Cl$_2$**.

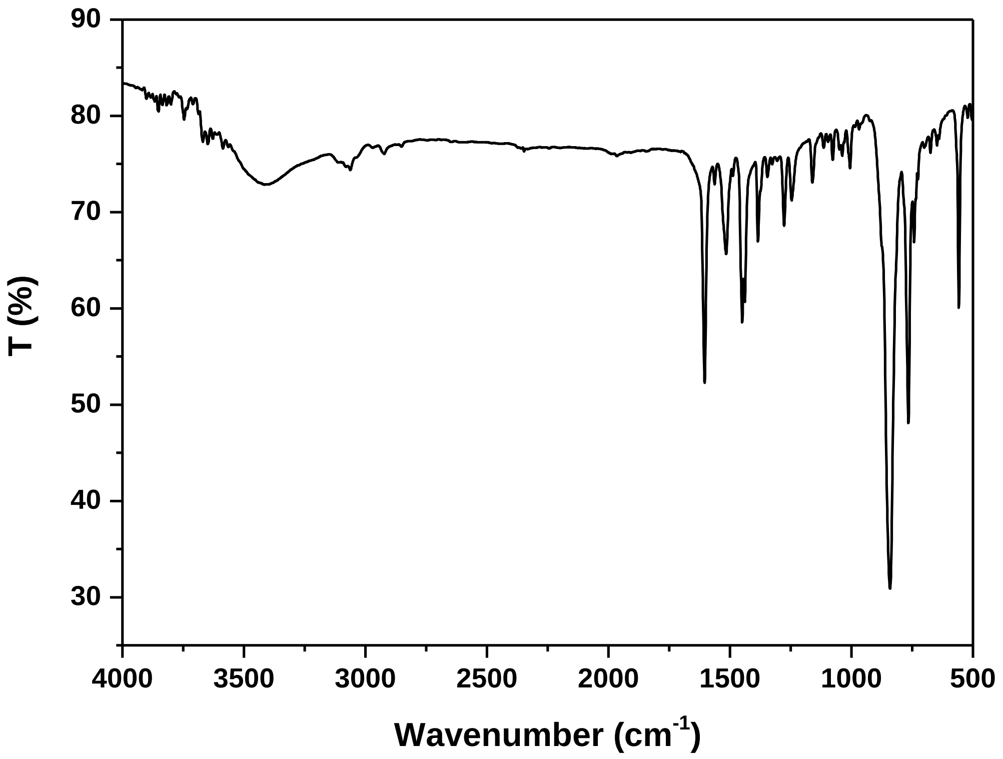

Figure S11. IR spectra (KBr) of {[Ru^{II}(trpy)]$_2$(μ-[Mn^{II}(Cl)$_2$(bpp)$_2$])}(PF$_6$)$_2$, **Ru$_2$Mn-Cl$_2$**.

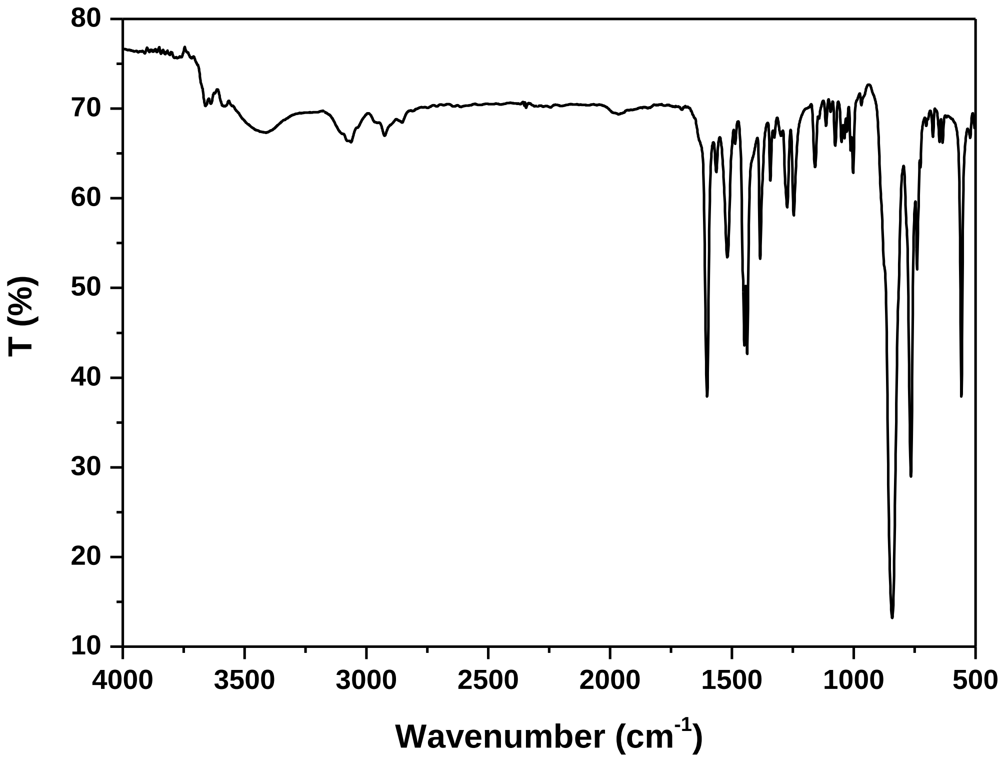

Figure S12. IR spectra (KBr) of {[Ru^{II}(trpy)]$_2$(μ-[Co^{II}(OAc)$_2$(bpp)$_2$])}(PF$_6$)$_2$, **Ru$_2$Co-OAc$_2$**.

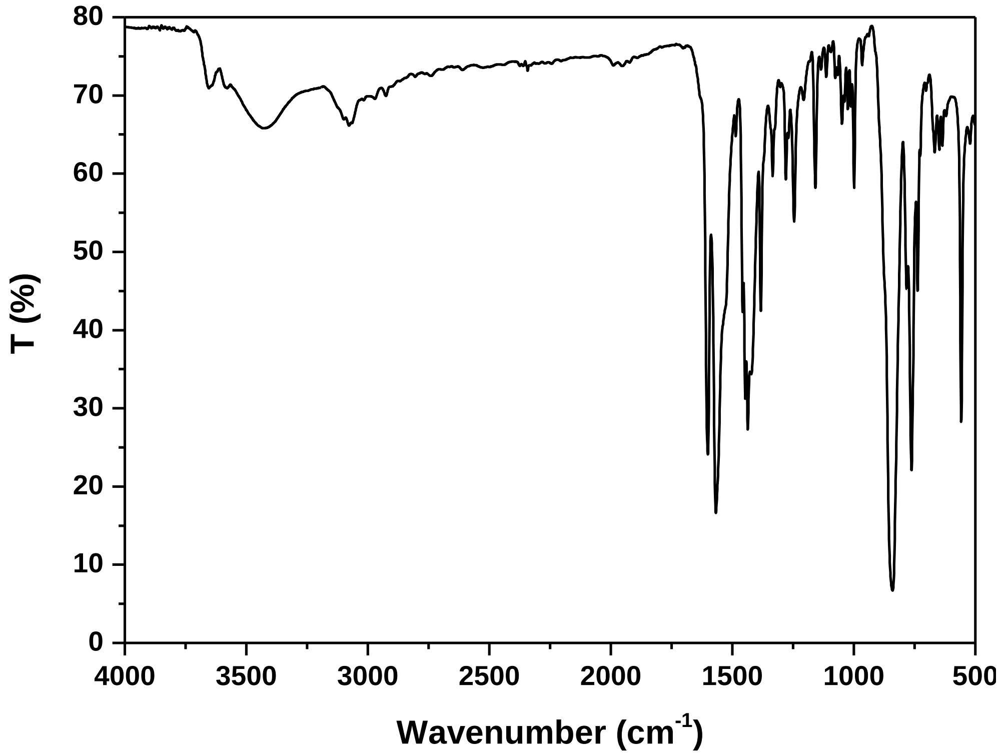

Figure S13. IR spectra (KBr) of {[Ru^{II}(trpy)]$_2$(μ-[Mn^{II}(OAc)$_2$(bpp)$_2$])}(PF$_6$)$_2$, **Ru$_2$Mn-OAc$_2$**.

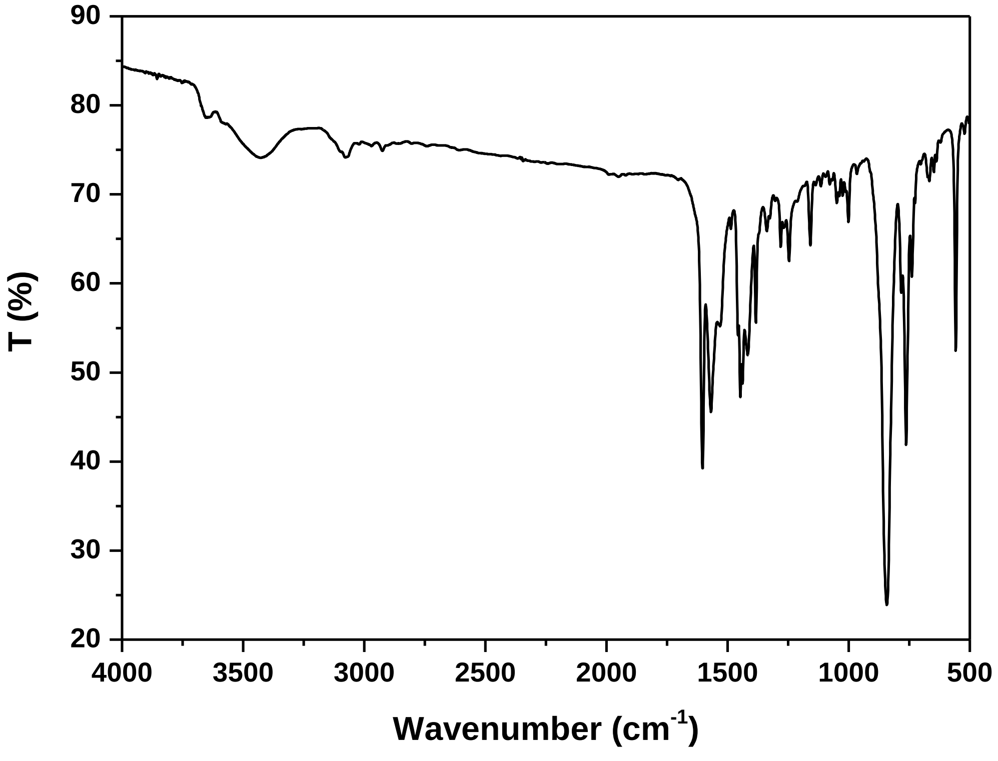

Figure S14. CV (black) and DPV (red) of {[Ru^{II}(trpy)]$_2$(μ-[Co^{II}(Cl)$_2$(bpp)$_2$])}(PF$_6$)$_2$, **Ru$_2$Co-Cl$_2$**, in CH_2Cl_2. All redox potentials in this and the subsequent figures are reported vs. NHE.

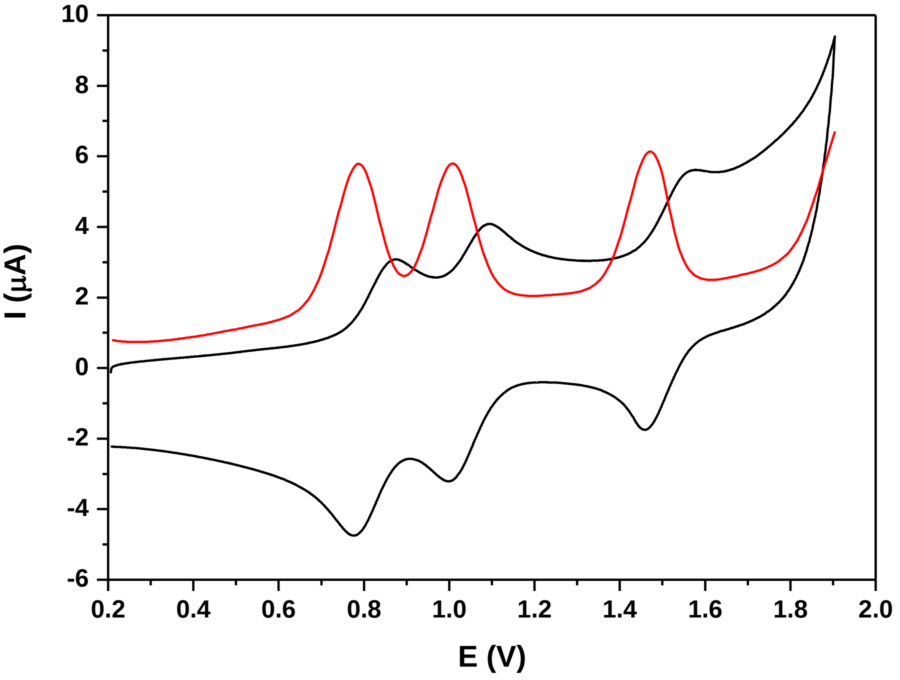

Figure S15. CV (black) and DPV (red) of {[Ru^{II}(trpy)]$_2$(μ-[Mn^{II}(Cl)$_2$(bpp)$_2$])}(PF$_6$)$_2$, **Ru$_2$Mn-Cl$_2$**, in CH_2Cl_2.

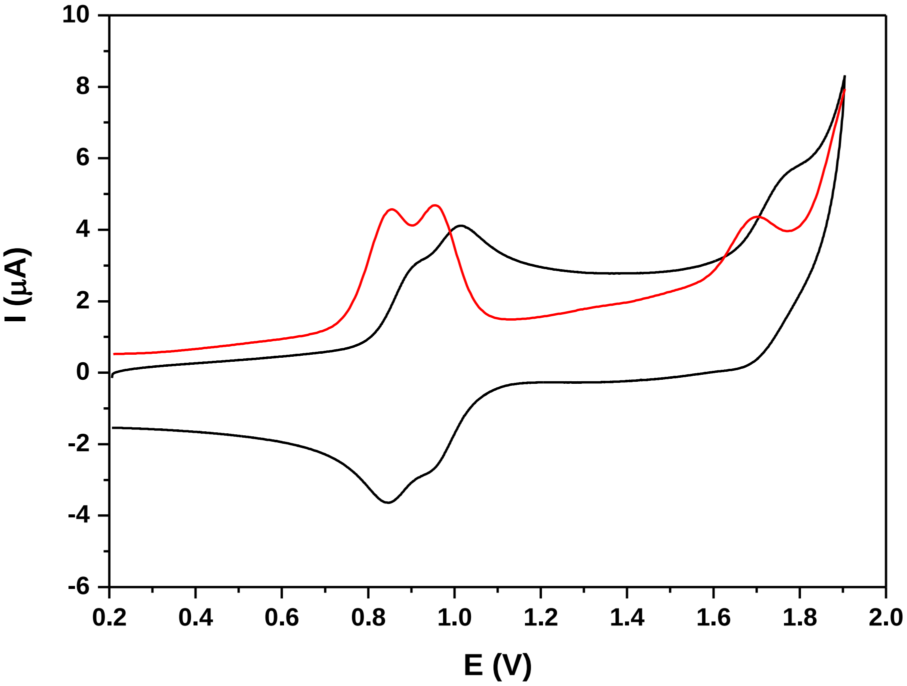

Figure S16. CV (black) and DPV (red) of {[Ru^{II}(trpy)]$_2$(μ-[Co^{II}(AcO)$_2$(bpp)$_2$])}(PF$_6$)$_2$, **Ru$_2$Co-OAc$_2$**, in CH$_3$CN.

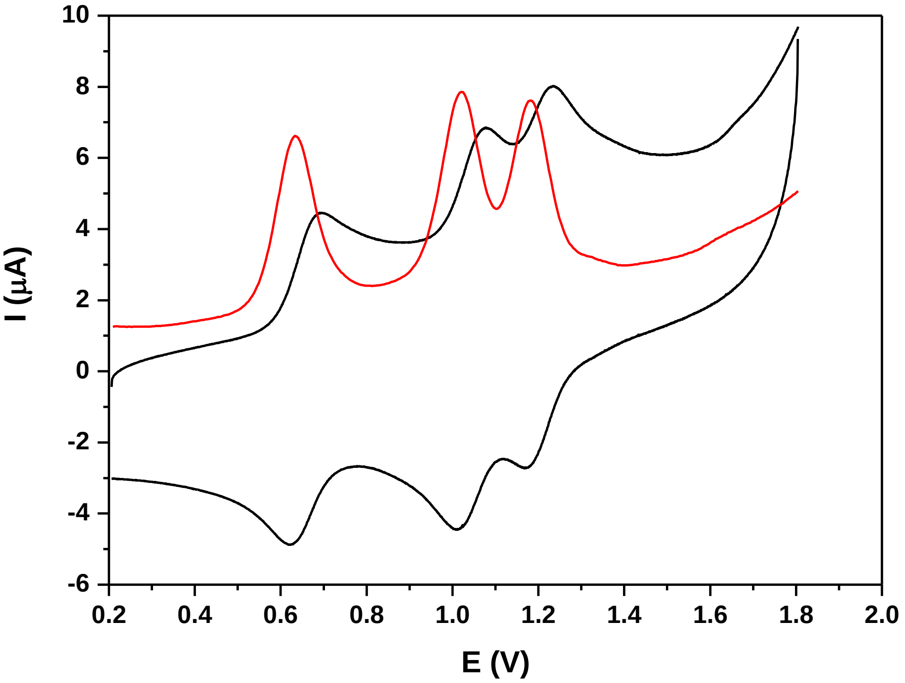

Figure S17. CV (black) and DPV (red) of {[Ru^{II}(trpy)]$_2$(μ-[Mn^{II}(AcO)$_2$(bpp)$_2$])}(PF$_6$)$_2$, **Ru$_2$Mn-OAc$_2$**, in CH$_3$CN.

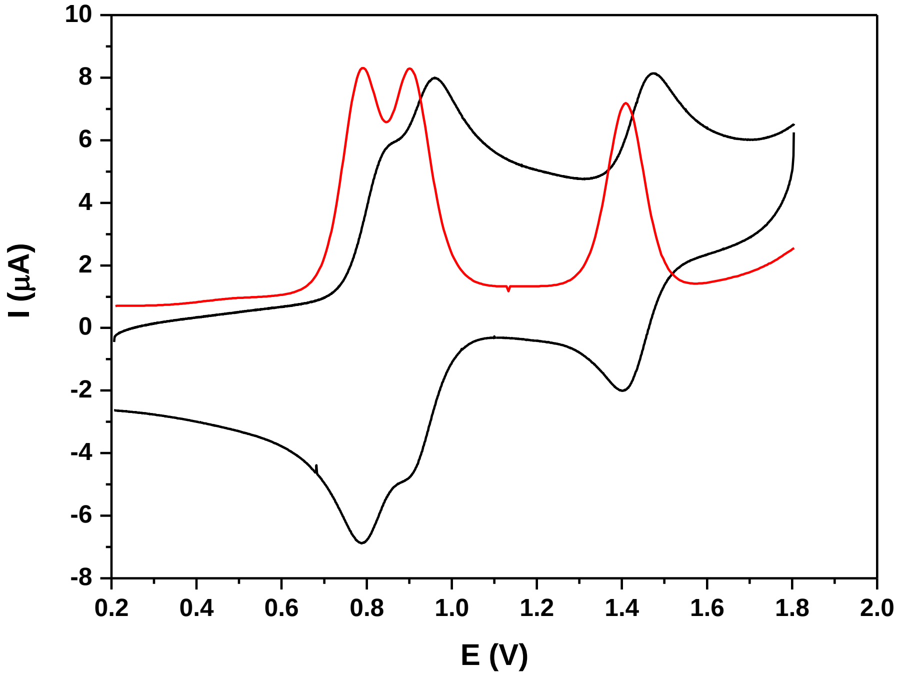

Figure S18. Cyclic voltammograms at a vitreous carbon electrode (diameter 3 mm) in CH_3CN, 0.1 M [(^{n}Bu$_4$N)ClO$_4$] of (A) a 0.25 mM solution of **Ru$_2$Co-OAc$_2$**, (B) after exhaustive oxidation at 0.85 V vs NHE (0.30 vs Ag/Ag$^+$) of the previous solution (formation of Ru$_2$(II)Co(III)), (C) after exhaustive oxidation at 1.40 vs NHE (0.85 vs Ag/Ag$^+$) of the previous solution (formation of Ru$_2$(III)Co(III)); scan rate 100 mV·s^{-1}. *E* vs NHE = *E* vs Ag/Ag$^+$ + 0.548.

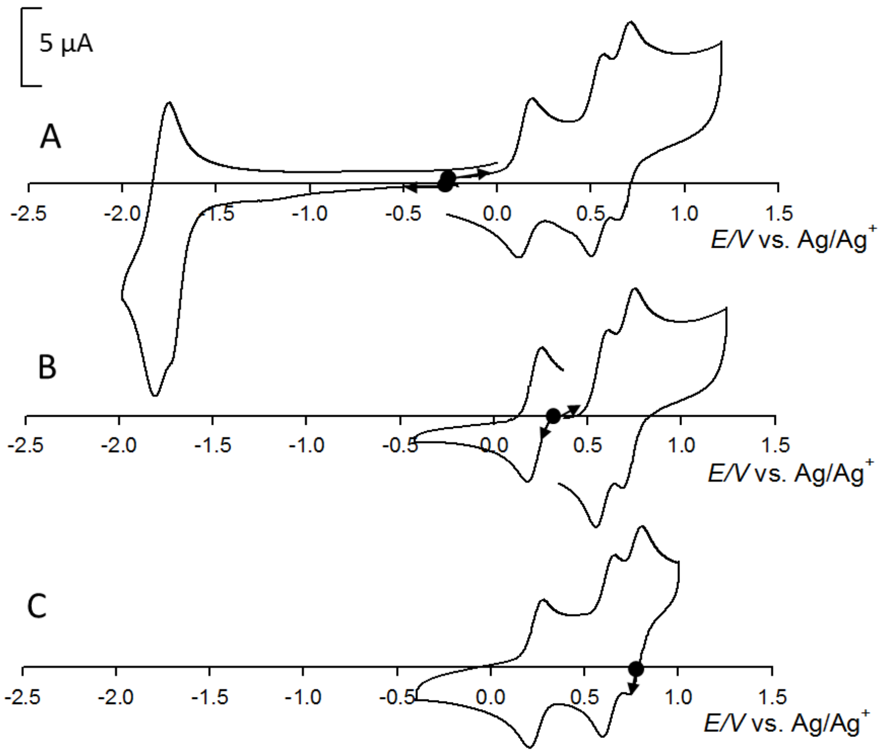

Figure S19. Voltammograms at a carbon Rotating Disk Electrode (2 mm diameter) of a 0.25 mM solution of **Ru$_2$Co-OAc$_2$** in CH_3CN, 0.1 M [(^{n}Bu$_4$N)ClO$_4$],: (A) initial solution, (B) after oxidation at 0.85 V vs NHE (0.30 vs Ag/Ag$^+$), (C) after oxidation at 1.40 vs NHE (0.85 vs Ag/Ag$^+$), rotation rate: ω = 600 rot/min, scan rate: v = 10 mVs^{-1}. *E* vs NHE = *E vs* Ag/Ag$^+$ + 0.548.

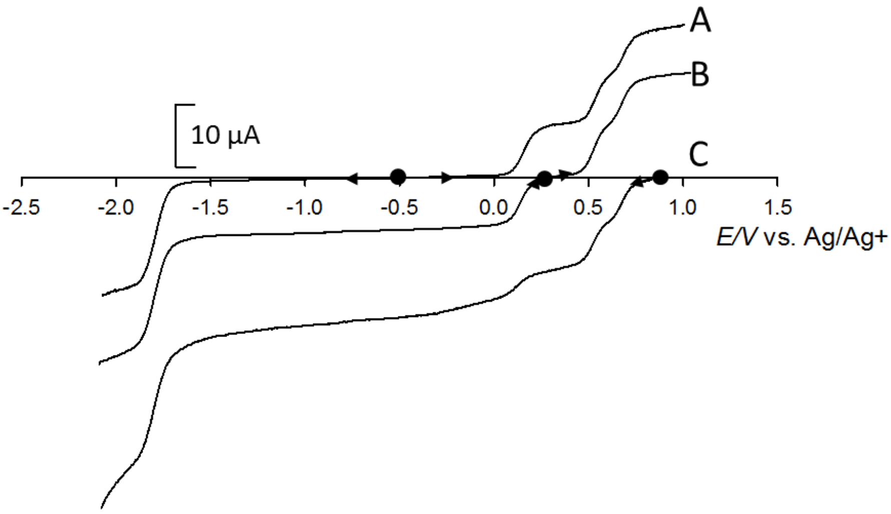

Figure S20. Cyclic voltammograms at a vitreous carbon electrode (diameter 3 mm) in CH_3CN, 0.1 M [($^nBu_4N$)ClO_4] of a 0.25 mM solution of **Ru_2Co-OAc_2** (black) and after two successive oxidations at 0.85 and 1.40 V vs NHE(0.30 and 0.85 V vs Ag/Ag^+), respectively, and one reduction at 0.35 V vs NHE (-0.20 V vs Ag/Ag^+) (red). *E* vs NHE = *E* vs Ag/Ag^+ + 0.548.

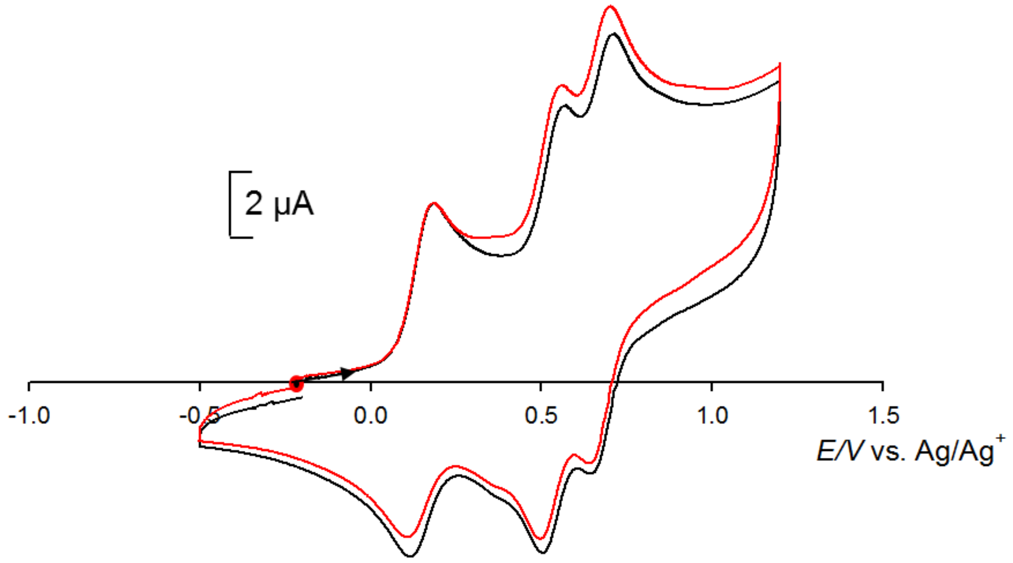

Figure S21. Cyclic voltammograms at a vitreous platinum electrode (diameter 5 mm) in CH_3CN, 0.1 M [($^nBu_4N$)ClO_4] of (A) a 0.41 mM solution of **Ru_2Mn-OAc$_2$**, (B) after exhaustive oxidation at 1.11 V vs NHE (0.56 vs Ag/Ag$^+$) of the previous solution (formation of Ru_2(III)Mn(II)), (C) after exhaustive oxidation at 1.69 vs NHE (1.14 vs Ag/Ag$^+$) of the previous solution (formation of Ru_2(III)Mn(III)); scan rate 50 mV·s^{-1}. *E* vs NHE = *E* vs Ag/Ag$^+$ + 0.548.

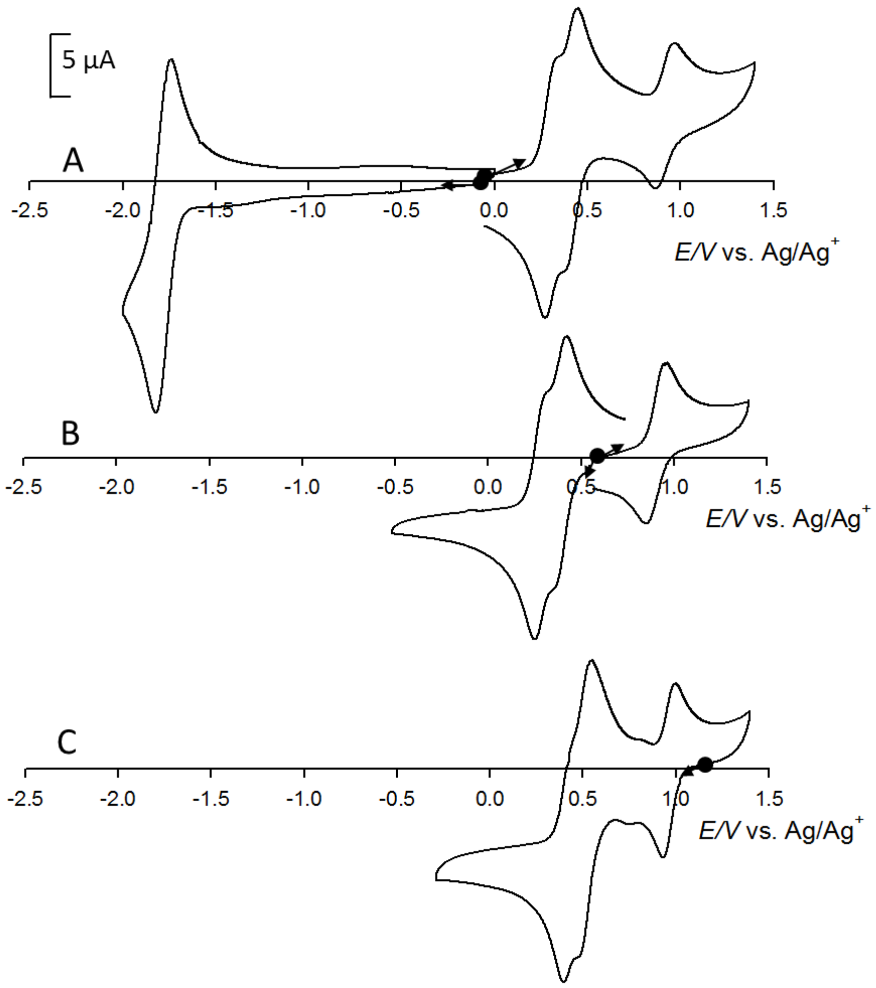

Figure S22. Cyclic voltammograms at a vitreous platinum electrode (diameter 5 mm) in CH_3CN, 0.1 M [(^{n}Bu_4N)ClO_4] of a 0.41 mM solution of **Ru_2Mn-OAc_2** (black) and after two successive oxidations at 1.11 and 1.69 V vs NHE (0.56 and 1.14 V vs Ag/Ag^+), respectively, and one reduction at 0.35 V vs NHE (-0.20 V vs Ag/Ag^+) (red). *E* vs NHE = *E* vs Ag/Ag^+ + 0.548

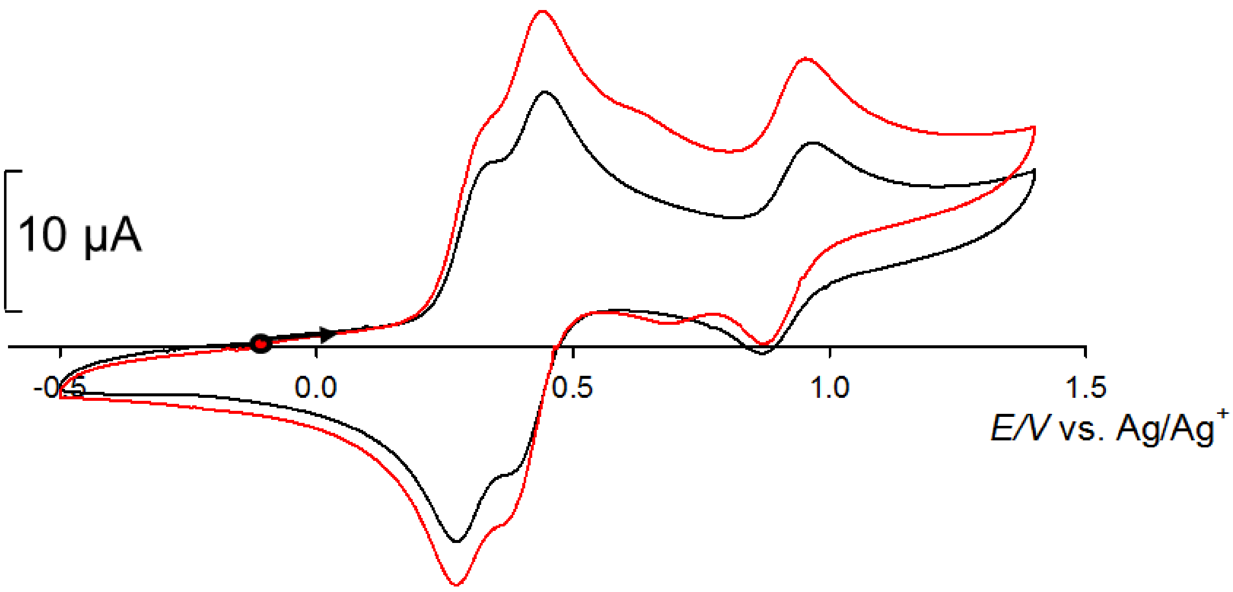

Figure S23. Reduced Magnetization *versus* field plots for compounds **Ru_2Mn-Cl_2** (black) and **Ru_2Mn-AcO_2** (red), measured at 2 K and variable field. Solid lines are just eye guides.

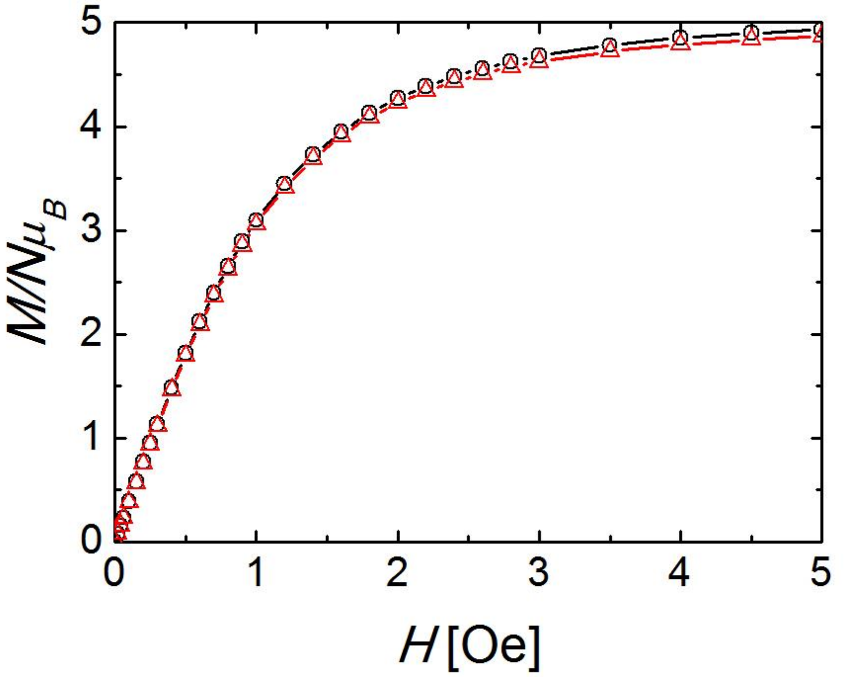

Figure S24. Isofield reduced Magnetization *versus* *H/T* plots for compounds **Ru_2Mn-Cl_2** (left) and **Ru_2Mn-AcO_2** (right), in the 1.8 to 6.8 K temperature range, for fields of 0.5, 1, 2, 3, 4 and 5 T.

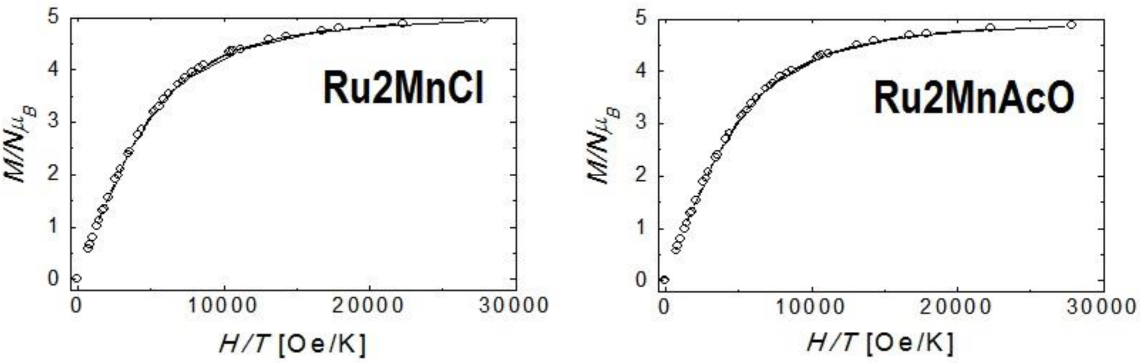

Figure S25. Reduced Magnetization *versus* field plots for compounds **Ru_2Co-Cl_2** (black) and **Ru_2Co-AcO_2** (red), measured at 2 K and variable field. Solid lines are just eye guides.

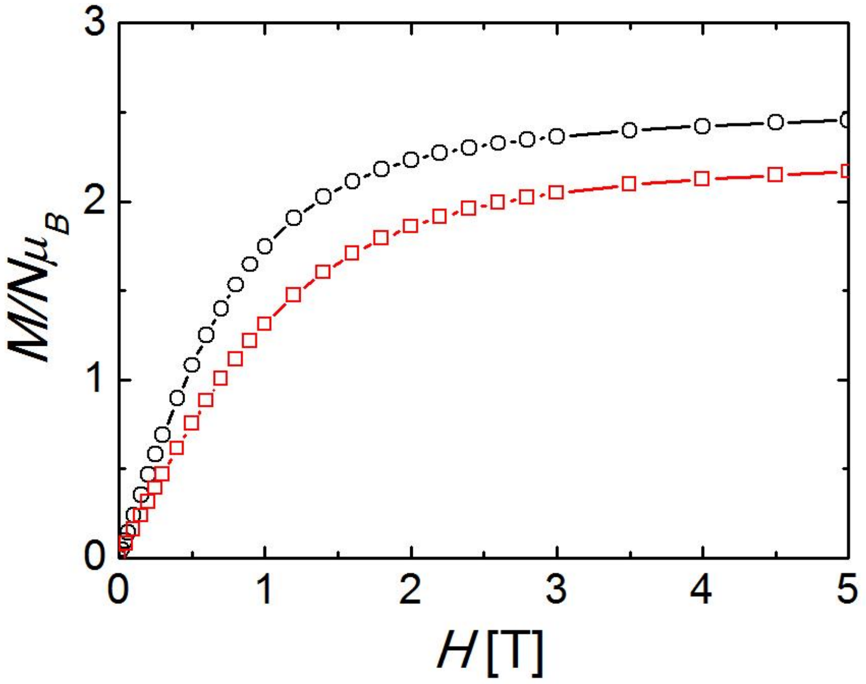

Figure S26. Isofield reduced magnetization *versus* H/T plots for compounds **Ru_2Co-Cl_2** (left) and **Ru_2Co-AcO_2** (right), in the 1.8 to 6.8 K temperature range, for fields of 0.5, 1, 2, 3, 4 and 5 T (only up to 4 T for **Ru_2Co-AcO_2**).

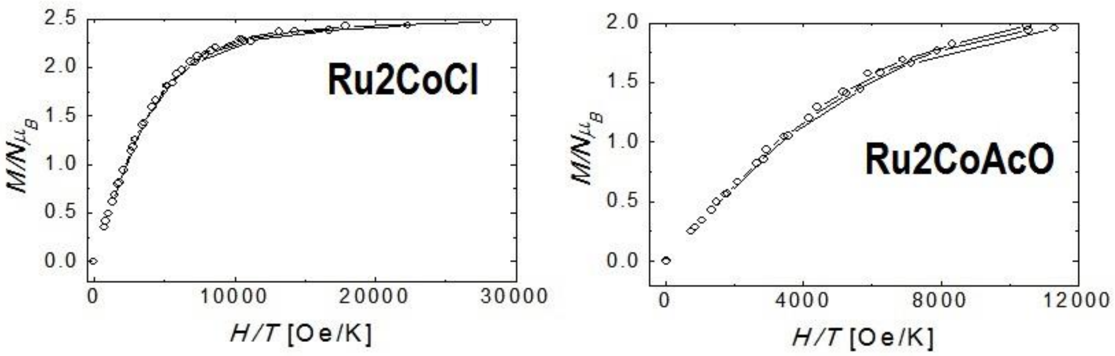

Figure S27. CV in a mixture (19:1) of pH = 1 CF_3SO_3H and CF_3CH_2OH, of (red) **Ru_2Co-OAc_2**, (black) **Ru_2Mn-OAc_2**, and (blue) ***in*-1**.

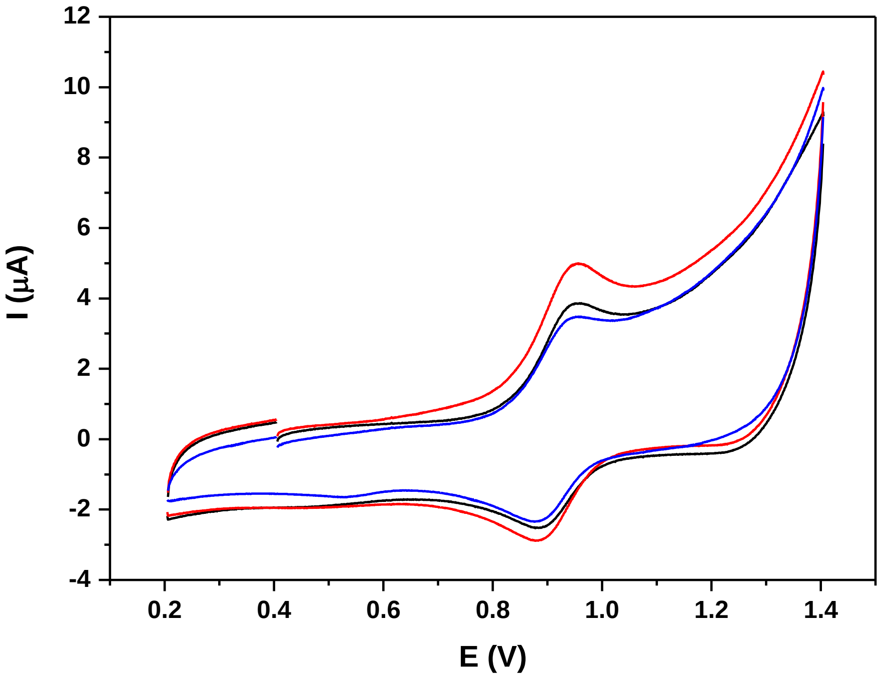

Figure S28. CV in a mixture (19:1) of pH = 7.0 (50 mM) phosphate buffer and CF_3CH_2OH, of (red) **Ru_2Co-OAc_2**, (black) **Ru_2Mn-OAc_2**, and (blue) ***in*-1**.

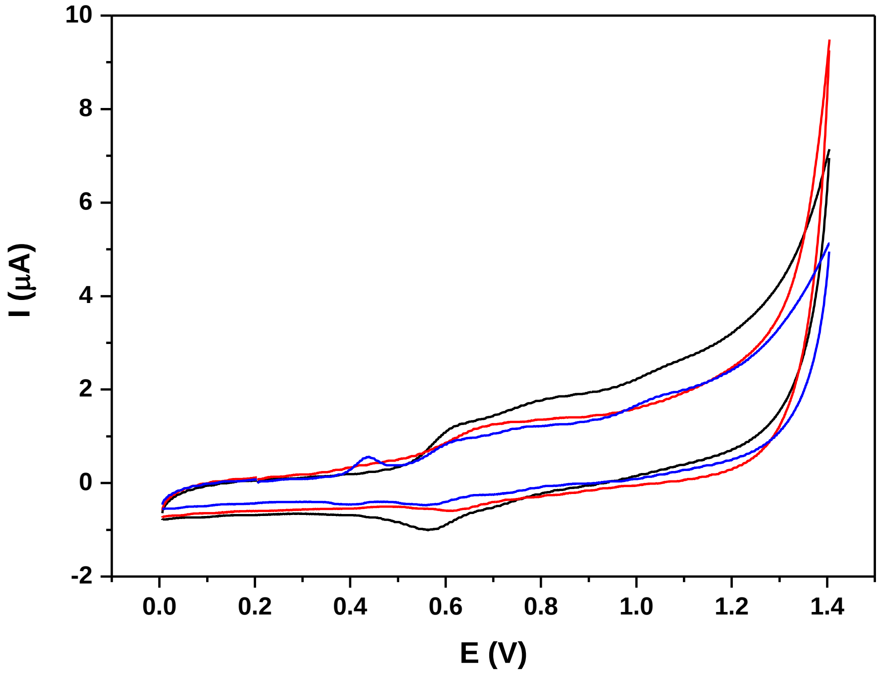

Figure S29. Photochemically induced oxidation of (black) **Ru_2Co-$(H_2O)_4$**, (red) $Co(CH_3COO)_2{\cdot}4H_2O$ and (blue) $[Ru(bpy)_3][ClO_4]_2$. Reaction conditions: $[catalyst]_f$ = 50 µM, $[[Ru(bpy)_3](ClO_4)_2]_f$ = 0.5 mM; $[Na_2S_2O_8]_f$ = 20 mM; total volume = 2 mL in a pH = 7.0 (50 mM) phosphate buffer solution and CF_3CH_2OH mixture (19:1). A 300 W Xenon lamp was used to illuminate the sample through a band pass filter of 440 nm at 298 K.

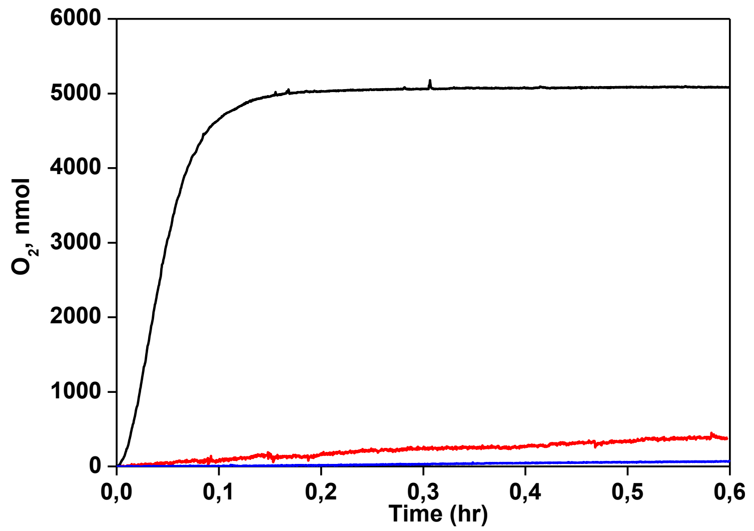

Figure S30. rRaman spectra of a mixture of 400 µLof a 0.5 M solution of Oxone in pH = 7.0 phosphate buffer mixed with 400 µL of $H_2{}^{16}O$ (red) and mixed with 400 µL of $H_2{}^{18}O$ (orange), after 30minutes (yellow), 1 hour (green), 2 hours (blue) and 10 hours (purple).

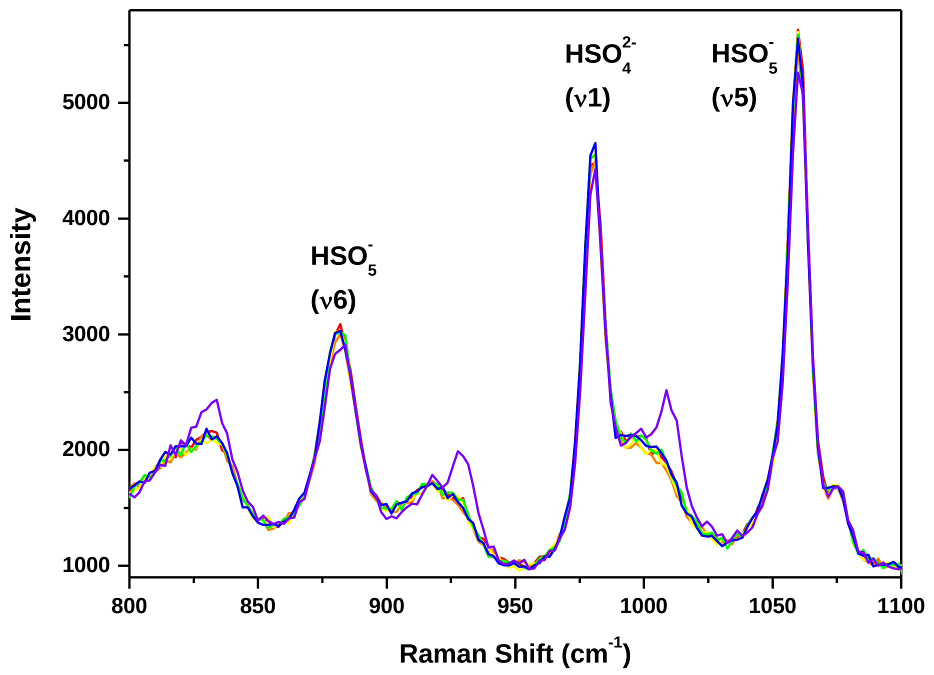

Figure S31. Ratios $^{32}O_2/^{34}O_2$ obtained from the on-line mass analysis. Top: isotopic labelling at 97% of $H_2^{18}O$. Bottom: isotopic labelling at 15% of $H_2^{18}O$.

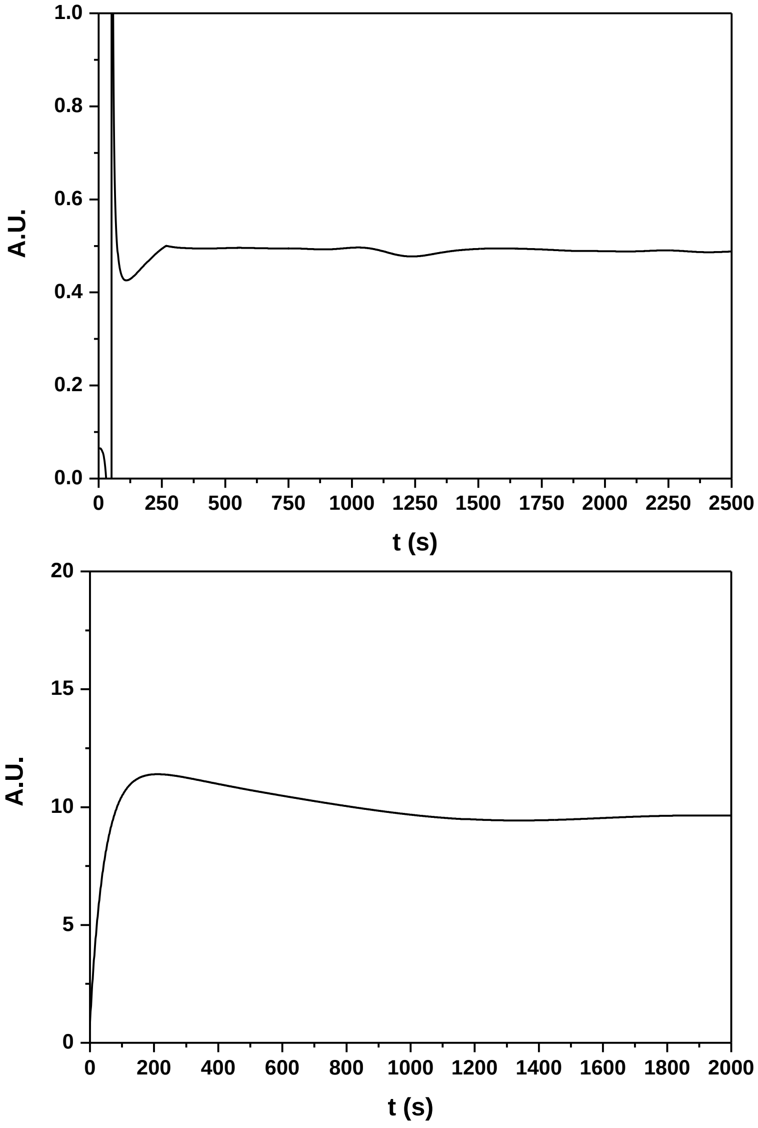

Supplement: Supplementary file 1 [file SC-007-C5SC04672F-s001.pdf]
